# Defining the optimal temporal and spatial resolution for cardiovascular magnetic resonance imaging feature tracking

**DOI:** 10.1186/s12968-021-00740-5

**Published:** 2021-05-17

**Authors:** Sören J. Backhaus, Georg Metschies, Marcus Billing, Jonas Schmidt-Rimpler, Johannes T. Kowallick, Roman J. Gertz, Tomas Lapinskas, Elisabeth Pieske-Kraigher, Burkert Pieske, Joachim Lotz, Boris Bigalke, Shelby Kutty, Gerd Hasenfuß, Sebastian Kelle, Andreas Schuster

**Affiliations:** 1 Department of Cardiology and Pneumology, University Medical Center Göttingen, Georg-August University, Robert-Koch-Str. 40, 37099 Göttingen, Germany; 2 German Center for Cardiovascular Research (DZHK), Partner Site Göttingen, Göttingen, Germany; 3 Institute for Diagnostic and Interventional Radiology, University Medical Center Göttingen, Georg-August University, Göttingen, Germany; 4 German Heart Center Berlin (DHZB), Department of Internal Medicine/Cardiology, University of Berlin, Charité Campus Virchow Clinic, Berlin, Germany; 5 DZHK (German Centre for Cardiovascular Research), Partner Site, Berlin, Germany; 6 Department of Cardiology, Medical Academy, Lithuanian University of Health Sciences, Kaunas, Lithuania; 7 Department of Cardiology and Pneumology, Charité Campus Benjamin Franklin, University Medical Center Berlin, Berlin, Germany; 8 Taussig Heart Center, Johns Hopkins Hospital, Baltimore, MD 21287 USA

**Keywords:** Myocardial deformation, Strain, Cardiovascular magnetic resonance, Temporal resolution, Spatial resolution, Reproducibility

## Abstract

**Background:**

Myocardial deformation analyses using cardiovascular magnetic resonance (CMR) feature tracking (CMR-FT) have incremental value in the assessment of cardiac function beyond volumetric analyses. Since guidelines do not recommend specific imaging parameters, we aimed to define optimal spatial and temporal resolutions for CMR cine images to enable reliable post-processing.

**Methods:**

Intra- and inter-observer reproducibility was assessed in 12 healthy subjects and 9 heart failure (HF) patients. Cine images were acquired with different temporal (20, 30, 40 and 50 frames/cardiac cycle) and spatial resolutions (high in-plane 1.5 × 1.5 mm through-plane 5 mm, standard 1.8 × 1.8 x 8mm and low 3.0 × 3.0 x 10mm). CMR-FT comprised left ventricular (LV) global and segmental longitudinal/circumferential strain (GLS/GCS) and associated systolic strain rates (SR), and right ventricular (RV) GLS.

**Results:**

Temporal but not spatial resolution did impact absolute strain and SR. Maximum absolute changes between lowest and highest temporal resolution were as follows: 1.8% and 0.3%/s for LV GLS and SR, 2.5% and 0.6%/s for GCS and SR as well as 1.4% for RV GLS. Changes of strain values occurred comparing 20 and 30 frames/cardiac cycle including LV and RV GLS and GCS (p < 0.001–0.046). In contrast, SR values (LV GLS/GCS SR) changed significantly comparing all successive temporal resolutions (p < 0.001–0.013). LV strain and SR reproducibility was not affected by either temporal or spatial resolution, whilst RV strain variability decreased with augmentation of temporal resolution.

**Conclusion:**

Temporal but not spatial resolution significantly affects strain and SR in CMR-FT deformation analyses. Strain analyses require lower temporal resolution and 30 frames/cardiac cycle offer consistent strain assessments, whilst SR measurements gain from further increases in temporal resolution.

**Supplementary Information:**

The online version contains supplementary material available at 10.1186/s12968-021-00740-5.

## Introduction

Myocardial deformation assessments have proven incremental value over sole volumetric approaches in the evaluation of cardiac function and risk prediction irrespective of modality and methodology, both in echocardiography [1, 2] and cardiovascular magnetic resonance (CMR) imaging [3–5]. Enhanced diagnostic accuracy has been demonstrated in the fields of heart failure (HF) with preserved (HFpEF) and reduced ejection fraction (HFrEF) [6–8], ischemic and non-ischemic cardiomyopathies [9, 10] as well as following acute myocardial infarction [11–13]. CMR represents the reference standard for the evaluation of cardiac morphology and function [14]. It allows the employment of various myocardial deformation assessments [15–17]. In contrast to the reference standard CMR tagging [18], CMR feature tracking (CMR-FT) can be applied on routinely acquired balanced steady state free precession (bSSFP) cine sequences [17] without the need for further deformation analyses sequences. CMR-FT has proven reliable reproducibility [19–23], being employed in a vast spectrum of cardiovascular diseases [9–11, 24]. Although CMR overcomes the limitations of anatomical plane restrictions as opposed to echocardiography [14, 25, 26], issues have been raised regarding through plane motion in 2 dimensional CMR-FT post-processing and regarding its lower temporal resolution compared to speckle-tracking echocardiography (STE) [26], especially since evidence from STE indicates a distinct impact of temporal resolution on strain assessment [27]. Despite the value of CMR-FT and increasing application, to date no recommendations exist for spatial and temporal resolutions in bSSFP cine images to allow for optimised post-processing. Consequently, this study aimed to determine the impact of temporal and spatial resolution on CMR-FT derived myocardial deformation indices and on their reproducibility and recommend imaging acquisition requirements for informed clinical use.

## Methods

### Cardiovascular magnetic resonance 

**Fig. 1:**
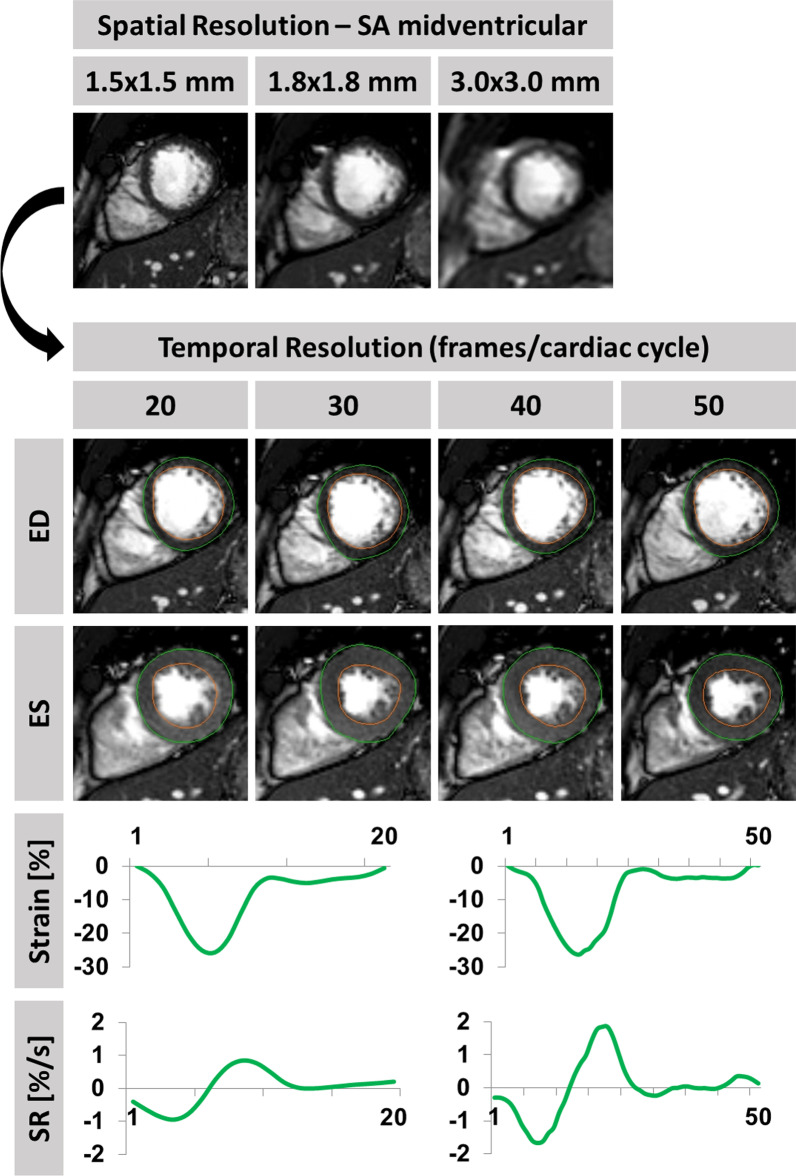
Strain and strain rate (SR) analyses with different spatial and temporal resolutions. Example of a healthy subject and end-diastolic mid left ventricular short axis views using high (1.5 × 1.5 mm in plane and 5 mm through-plane), standard (1.8 × 1.8 mm in plane and 8 mm through-plane) and low (3.0 × 3.0 mm in plane and 10 mm through-plane) spatial resolutions. Below different temporal resolutions of 20, 30, 40 and 50 frames/cardiac cycle in high spatial resolution (1.5 × 1.5 mm in plane and 5 mm through-plane) with traced borders at end-diastole (ED) and end-systole (ES) are displayed. Strain and strain rate (SR) curves for global circumferential strain (GCS) are exemplary shown for high spatial with either lowest (20) or highest (50) temporal resolution. Final strain values are based on endocardial strain only

The study population consisted of 12 healthy subjects and 9 patients with known ischemic heart disease and symptoms of HF. The study was approved by the Ethics Committee of the Charité-University Medicine Berlin and complied with the Declaration of Helsinki. All individuals gave written informed consent before participating in the study. Prospective electrocardiogram (ECG)-gated bSSFP cine sequences were acquired on a clinical 1.5 T CMR scanner (Achieva, Philips Healthcare, Best, the Netherlands) for long-axis 2 chamber (2Ch)- and 4-chamber (4Ch) views as well as a short axis stack. Imaging parameters included temporal resolutions of 20, 30, 40 and 50 frames/cardiac cycle. The reconstructed cardiac phases were obtained using a fixed interpolation factor of 50%. Spatial resolutions comprised high (1.5 × 1.5 mm in plane and 5 mm through-plane), standard (1.8 × 1.8 mm in plane and 8 mm through-plane) as well as low (3.0 × 3.0 mm in plane and 10 mm through-plane) settings (Fig. 1). For the purposes of this manuscript, the resolutions are referred to by their through-plane value of 5 mm, 8 mm and 10 mm respectively.

### Feature-tracking

CMR-FT was performed using the commercially available software QStrain (version 3.1.16.9, Medis Medical Imaging Systems, Leiden, Netherlands, Fig. 1 and Additional file 1: Figure S1) for left ventricular (LV) global as well as segmental longitudinal and circumferential strain as well as associated systolic strain rates (GLS/GCS GLS SR/ GCS SR) and right ventricular (RV) global longitudinal strains (RV GLS). LV and RV were tracked at endocardial borders. The LV was tracked in long axis (2Ch and 4Ch) and short axis orientations. The RV was tracked in the 4Ch only [21]. Contours were manually traced in end-diastole and end-systole, the tracking algorithm was then applied following the tissue features over the cardiac cycle. Strain is reported as the percentage value of absolute shortening (Strain [%] = $\frac{Length\;\text{endsystole }-Length\;\text{enddiastole}}{Length\;\text{enddiastole}}$ * 100). Accuracy was visually reviewed, where appropriate corrections were made by the observer to the initial contours only. Short axis strains (GCS) were assessed at basal (requiring circular myocardium and the absence of LV outflow tract during the cardiac cycle), midventricular (with both papillary muscles visible) and apical (maintained blood-pool throughout the cardiac cycle) positions. Intra-observer and inter-observer reproducibility was calculated within two operators in a core-laboratory with proven excellent intra- and inter-observer reproducibility in previous trials [21, 22, 28]. Both observers were well experienced in CMR and received dedicated training in CMR. Observer one performed two rounds 4 weeks apart for the assessment of intra-observer reproducibility, observer two performed one round for the assessment of inter-observer reproducibility respectively. Each round consisted of 3 repeated measurements, final strain values were based on the average to enhance reproducibility and decrease variability [21, 22]. Reproducibility of GLS and GCS was assessed on segmental level based on the segments proposed in the American Heart Association 16 segments model [29].

### Statistics

Intra- and inter-observer variability was evaluated based on Bland–Altman analyses [mean difference between measurements and corresponding 95% confidence interval (CI)] [30], intra-class correlation coefficients (ICC) for absolute agreements and the coefficient of variation (CoV, SD of mean difference divided by the mean $\frac{SD (MD)}{mean}$) [20]. An ICC > 0.74 was considered excellent, good between 0.60 and 0.74, fair between 0.4 and 0.59 and poor below 0.4. Continuous variables are expressed as mean ± standard deviation (SD). Dependent variables were tested using the Wilcoxon signed-rank test. An alpha level of 0.05 was considered statistically significant. Statistical analyses were performed using SPSS (version 24 for Windows, Statistical Package for the Social Sciences, International Business Machines, Inc., Armonk, New York, USA) and Excel (Microsoft Corporation, Redmond, Washington, USA).

## Results

### Demographics

The study population comprised 12 healthy subjects and 9 HF patients. Healthy subjects had a median age of 29 years (IQR 25, 34) with normal LV ejection fraction (LVEF) (median 60%; IQR 59, 60). HF patients had a median age of 67 years (ICR 51, 69) with LVEF (median 56%; IQR 47, 57). The median heart rate during CMR acquisition was 66/min (IQR 60, 71). LV strain and SR as well as RV strain values are reported in association to spatial and temporal resolution in Table 1.

**Table 1:** Cardiac function patients

Resolution		Cardiac function									
Spatial (mm)	Temporal (frames/cycle)	LV GLS (%)		LV GLS SR (%/s)		GCS (%)		GCS SR (%/s)		RV GLS (%)	
5	20	− 20.4 (3.66)		− 0.77 (0.16)		− 28.3 (5.51)		− 1.23 (0.34)		− 24.2 (3.50)	
5	30	− 21.3 (3.80)	**0.017**	− 0.94 (0.21)	** < 0.001**	− 30.1 (6.05)	** < 0.001**	− 1.57 (0.41)	** < 0.001**	− 25.6 (4.43)	**0.046**
5	40	− 21.5 (3.84)	0.244	− 1.01 (0.23)	**0.001**	− 30.6 (5.86)	0.274	− 1.70 (0.41)	**0.001**	− 25.4 (4.53)	0.741
5	50	− 21.5 (3.86)	0.903	− 1.07 (0.23)	**0.008**	− 30.8 (5.81)	0.627	− 1.80 (0.38)	**0.013**	− 25.4 (4.20)	0.903
8	20	− 21.0 (3.60)		− 0.81 (0.17)		− 28.6 (5.28)		− 1.25 (0.32)		− 24.2 (4.59)	
8	30	− 20.9 (4.09)	0.741	− 0.95 (0.20)	** < 0.001**	− 29.1 (4.98)	**0.015**	− 1.59 (0.38)	** < 0.001**	− 24.8 (4.68)	0.170
8	40	− 20.7 (3.40)	0.768	− 0.98 (0.20)	0.117	− 29.8 (6.01)	0.205	− 1.67 (0.39)	**0.001**	− 25.2 (4.50)	0.455
8	50	− 21.3 (4.10)	0.339	− 1.05 (0.20)	**0.001**	− 30.3 (5.76)	0.217	− 1.73 (0.39)	**0.003**	− 25.4 (3.80)	0.986
10	20	− 20.7 (4.29)		− 0.82 (0.21)		− 28.2 (5.80)		− 1.22 (0.37)		− 22.8 (4.45)	
10	30	− 22.0 (4.17)	**0.001**	− 0.99 (0.21)	** < 0.001**	− 29.6 (5.82)	0.917	− 1.59 (0.46)	** < 0.001**	− 23.7 (3.78)	0.099
10	40	− 22.0 (4.67)	0.768	− 1.05 (0.25)	**0.030**	− 30.5 (5.80)	0.085	− 1.72 (0.46)	**0.014**	− 24.1 (3.92)	0.590
10	50	− 22.5 (4.55)	0.062	− 1.11 (0.26)	**0.002**	− 30.5 (5.77)	0.002	− 1.76 (0.44)	**0.014**	− 24.2 (3.92)	0.601

Continuous variables are expressed as mean (standard deviation). *LV/RV* left/right ventricular, *GLS/GCS/GRS* global longitudinal/circumferential/radial strain, *SR* systolic strain rate. Wilcoxon-signed rank test was used to determine statistical significance. P-values were calculated comparing one temporal resolution to the successive higher one. Bold p-values indicate statistical significance

### Cardiac function

#### Temporal resolution

**Fig. 2:**
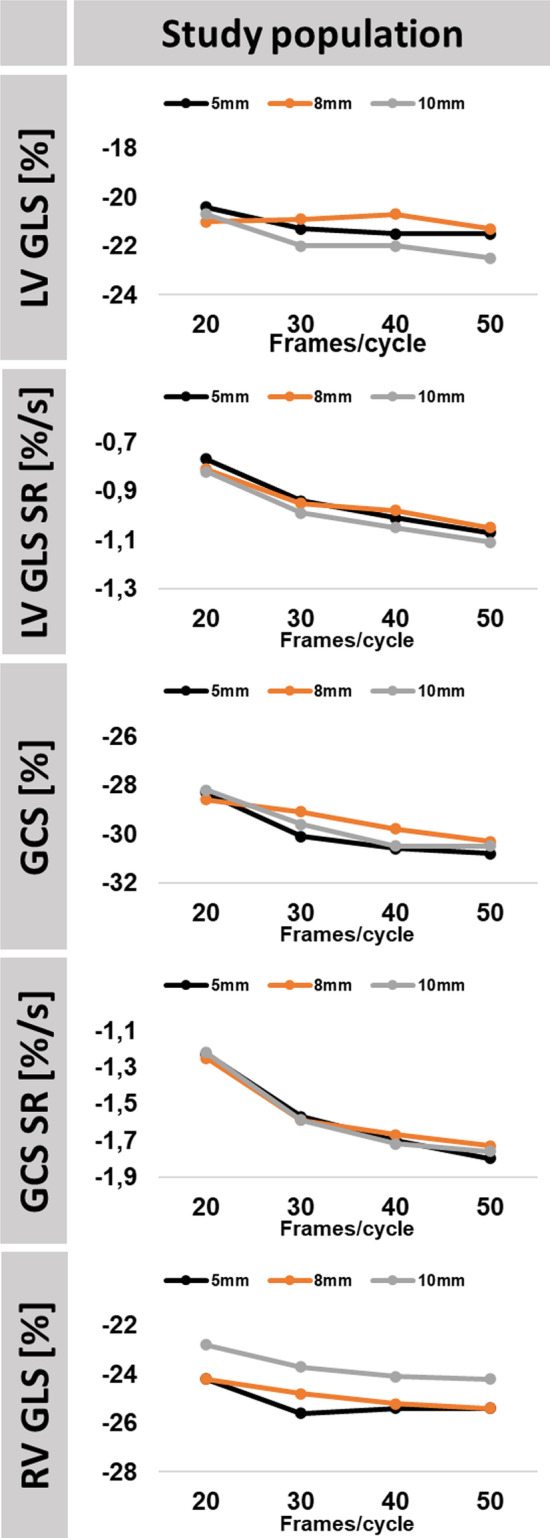
Impact of Temporal Resolution on Strain and Strain Rate (SR). The graph shows absolute strain and SR values obtained in deformation imaging in relation to temporal (20/30/40/50 frames/cardiac cycle) and spatial resolution (5, 8 and 10 mm) for healthy subjects and heart failure patients. LV/RV: left/right ventricle, *GLS* global longitudinal strain, *GCS* global circumferential strain, *SR* strain rate

Increasing temporal resolution was associated with increasing absolute strain and SR (Table 1 and Fig. 2). The lowest temporal resolution was associated with the lowest absolute strain and SR. Absolute changes between 0.3 and 1.8% for LV GLS and 0.24–0.30%/s for associated GLS SR, 1.7–2.5% for GCS and 0.48–0.57%/s for associated GCS SR and 1.2–1.4% for RV GLS were observed comparing the lowest to the highest temporal resolution.

Significant changes in strain analyses were observed exclusively comparing the low temporal resolutions of 20 to 30 frames/cardiac cycle at a spatial resolution of 5 mm (LV p = 0.017 and RV GLS p = 0.046 as well as GCS p < 0.001), 8 mm (GCS p = 0.015) and 10 mm (LV GLS p = 0.001). In contrast, significant changes occurred up to the highest temporal resolution for SR (LV GLS and GCS SR) measurements and amongst all spatial resolutions (Table 1).

#### Spatial resolution

Consistent absolute strain and SR values were obtained irrespective of different spatial resolutions.

### Reproducibility

**Fig. 3:**
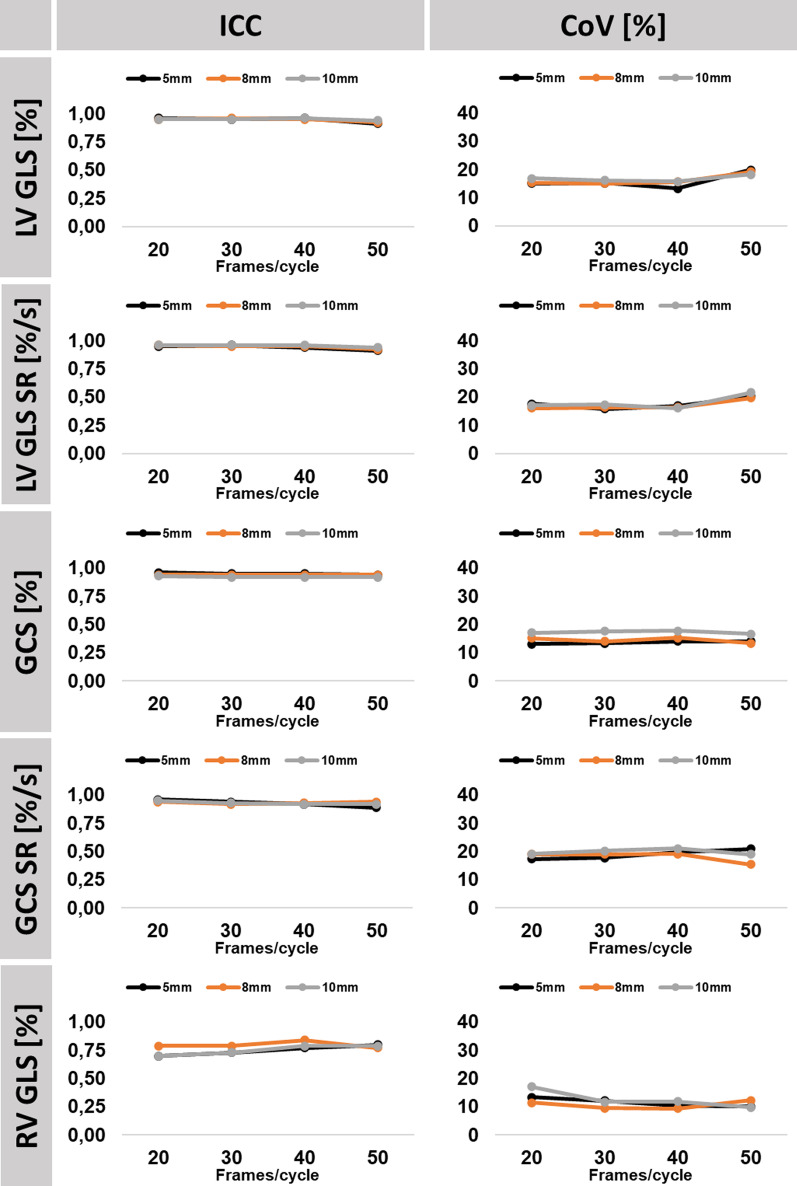
Intraobserver reproducibility. The graph shows intraclass correlation coefficients (ICC) and coefficients of variation (CoV) for intra-observer reproducibility in association to temporal (20/30/40/50 frames/cardiac cycle) and spatial resolution (5, 8 and 10 mm). *LV/RV* left/right ventricle, *GLS* global longitudinal strain, *GCS* global circumferential strain, *SR* strain rate

**Fig. 4:**
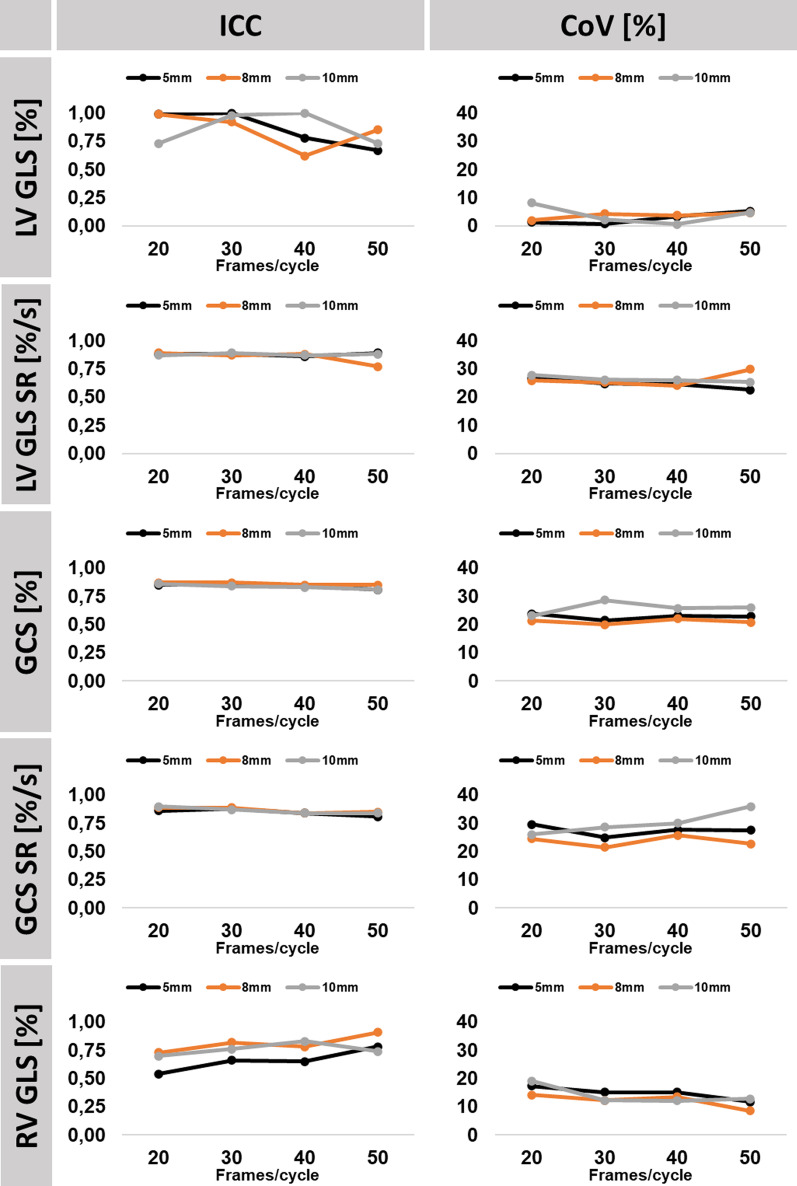
Interobserver reproducibility. The graph shows interclass correlation coefficients (ICC) and coefficients of variation (CoV) for inter-observer reproducibility in association to temporal (20/30/40/50 frames/cardiac cycle) and spatial resolution (5, 8 and 10 mm). *LV/RV* left/right ventricle, *GLS* global longitudinal strain, *GCS* global circumferential strain, *SR* strain rate

Mean differences as well as corresponding SD, ICC and CoV of assessed strain and SR values are reported in Figs. 3 and 4 as well as in the supplements for LV GLS (Table 2), LV GLS SR (Table 3), LV GCS (Table 4), LV GCS SR (Table 5) and RV GLS (Additional file 1: Table S1) and Additional file 1: Figures S2–S13. Reproducibility on segmental level was excellent for LV GLS and GCS (ICC ≥ 0.81) as well as associated strain rates (ICC ≥ 0.77). Reproducibility in segments with wall motion abnormalities was good to excellent for GLS and GCS (ICC ≥ 0.73) and associated strain rates (ICC ≥ 0.82) (Additional file 1: Tables S2–S5).

**Table 2:** Left ventricular GLS intra- and inter-observer reproducibility

Resolution		Intra-Observer			Inter-observer		
Spatial (mm)	Temporal (frames/cycle)	Mean difference (SD of the Diff.)	ICC (95% CI)	CoV (%)	Mean difference (SD of the Diff.)	ICC (95% CI)	CoV (%)
5	20	0.13 (3.11)	0.96 (0.95–0.97)	15.0	0.36 (5.10)	0.87 (0.83–0.90)	24.8
5	30	0.05 (3.36)	0.95 (0.94–0.96)	15.2	0.62 (5.34)	0.86 (0.82–0.89)	24.6
5	40	0.27 (3.00)	0.96 (0.95–0.97)	13.3	0.59 (5.31)	0.86 (0.83–0.89)	24.0
5	50	0.38 (4.55)	0.91 (0.89–0.93)	19.8	0.23 (5.31)	0.87 (0.83–0.90)	23.4
8	20	0.55 (3.31)	0.95 (0.94–0.96)	15.3	0.90 (5.14)	0.86 (0.82–0.89)	24.6
8	30	0.18 (3.26)	0.96 (0.95–0.97)	15.0	0.83 (5.53)	0.86 (0.82–0.89)	26.0
8	40	0.51 (3.43)	0.95 (0.93–0.96)	15.6	0.16 (5.58)	0.85 (0.81–0.88)	25.7
8	50	0.70 (4.48)	0.92 (0.90–0.94)	19.2	0.59 (5.35)	0.88 (0.84–0.90)	23.6
10	20	0.54 (3.57)	0.95 (0.94–0.96)	16.8	0.44 (5.30)	0.89 (0.86–0.91)	25.6
10	30	0.36 (3.72)	0.95 (0.94–0.96)	16.2	0.69 (5.96)	0.87 (0.83–0.90)	26.5
10	40	0.02 (3.64)	0.96 (0.95–0.97)	15.7	0.75 (6.23)	0.86 (0.81–0.89)	27.3
10	50	0.00 (4.37)	0.94 (0.93–0.96)	18.2	1.04 (5.94)	0.88 (0.84–0.91)	25.5

*SD* standard deviation, *Diff* difference, *ICC* intraclass correlation coefficient, *CoV* coefficient of variation, *LV* left ventricular, *GLS* global longitudinal strain (n = 252 data points)

**Table 3:** Left ventricular GLS SR intra- and inter-observer reproducibility

Resolution		Intra-observer			Inter-Observer		
Spatial (mm)	Temporal (frames/cycle)	Mean difference (SD of the Diff.)	ICC (95% CI)	CoV (%)	Mean difference (SD of the Diff.)	ICC (95% CI)	CoV (%)
5	20	0.01 (0.15)	0.95 (0.94–0.96)	17.6	0.01 (0.23)	0.88 (0.84–0.90)	26.7
5	30	0.00 (0.18)	0.96 (0.95–0.97)	15.8	0.02 (0.28)	0.88 (0.85–0.91)	24.9
5	40	0.02 (0,22)	0.94 (0.93–0.96)	16.9	0.00 (0.32)	0.86 (0.83–0.89)	25.4
5	50	0.06 (0.30)	0.91 (0.89–0.93)	20.2	0.01 (0.33)	0.89 (0.86–0.91)	22.5
8	20	0.03 (0.15)	0.96 (0.95–0.97)	16.0	0.03 (0.22)	0.89 (0.86–0.92)	25.0
8	30	0.00 (0.19)	0.95 (0.94–0.96)	16.3	0.03 (0.28)	0.87 (0.84–0.90)	25.4
8	40	0.02 (0.20)	0.95 (0.93–0.96)	16.4	0.00 (0.29)	0.88 (0.84–0.90)	24.2
8	50	0.03 (0.30)	0.92 (0.90–0.94)	19.6	0.02 (0.49)	0.77 (0.70–0.82)	32.9
10	20	0.02 (0.15)	0.96 (0.95–0.97)	17.0	0.02 (0.26)	0.87 (0.83–0.90)	29.2
10	30	0.03 (0.21)	0.96 (0.94–0.97)	17.3	0.01 (0.32)	0.89 (0.85–0.91)	27.1
10	40	0.02 (0.21)	0.96 (0.95–0.97)	16.0	0.01 (0.35)	0.87 (0.84–0.90)	27.0
10	50	0.05 (0.33)	0.94 (0.92–0.95)	21.6	0.02 (0.38)	0.88 (0.85–0.91)	25.7

*SD* standard deviation, *Diff* difference, *ICC* intraclass correlation coefficient, *CoV* coefficient of variation, *LV* left ventricular, *SRs* systolic strain rate (n = 252 data points)

**Table 4:** Left ventricular GCS intra- and inter-observer reproducibility

Resolution		Intra-observer			Inter-observer		
Spatial (mm)	Temporal (frames/cycle)	Mean difference (SD of the Diff.)	ICC (95% CI)	CoV (%)	Mean difference (SD of the Diff.)	ICC (95% CI)	CoV (%)
5	20	0.14 (3.69)	0.96 (0.95–0.97)	13.1	1.16 (6.55)	0.85 (0.81–0.88)	23.8
5	30	0.22 (4.02)	0.95 (0.94–0.96)	13.3	1.91 (6.27)	0.86 (0.82–0.90)	21.5
5	40	0.18 (4.31)	0.95 (0.93–0.96)	14.1	1.92 (6.85)	0.84 (0.79–0.88)	23.1
5	50	0.19 (4.39)	0.94 (0.92–0.95)	14.1	1.56 (6.90)	0.81 (0.76–0.85)	22.9
8	20	0.51 (4.29)	0.94 (0.93–0.95)	15.1	1.25 (5.90)	0.87 (0.83–0.90)	21.4
8	30	0.41 (4.08)	0.94 (0.93–0.95)	14.0	1.00 (5.68)	0.87 (0.84–0.90)	20.0
8	40	0.57 (4.66)	0.94 (0.92–0.95)	15.3	1.42 (6.49)	0.85 (0.81–0.88)	22.0
8	50	0.76 (4.10)	0.94 (0.93–0.96)	13.4	1.37 (6.16 =	0.85 (0.81–0.88)	20.8
10	20	0.36 (4.79)	0.93 (0.91–0.94)	17.1	0.74 (6.33)	0.86 (0.83–0.89)	23.1
10	30	0.23 (5.22)	0.92 (0.91–0.94)	17.6	1.71 (7.16)	0.84 (0.79–0.87)	25.0
10	40	0.61 (5.43)	0.92 (0.90–0.94)	17.8	1.19 (7,63)	0.83 (0.79–0.86)	25.7
10	50	0.58 (5.11)	0.92 (0.90–0.94)	16.7	0.83 (7.77)	0.81 (0.76–0.85)	26.0

*SD* standard deviation, *Diff* difference, *ICC* intraclass correlation coefficient, *CoV* coefficient of variation, *LV* left ventricular, *GCS* global circumferential strain (n = 336 data points)

**Table 5:** LV GCS SR intra- and inter-observer reproducibility

Resolution		Intra-observer			Inter-observer		
Spatial (mm)	Temporal (frames/cycle)	Mean difference (SD of the Diff.)	ICC (95% CI)	CoV (%)	Mean difference (SD of the Diff.)	ICC (95% CI)	CoV (%)
5	20	0.04 (0.22)	0.96 (0.95–0.97)	17.3	0.02 (0.37)	0.86 (0.83–0.89)	29.6
5	30	0.07 (0.31)	0.94 (0.92–0.95)	17.8	0.04 (0.42)	0.88 (0.85–0.90)	25.0
5	40	0.09 (0.38)	0.92 (0.90–0.94)	19.7	0.01 (0.52)	0.84 (0.80–0.87)	27.7
5	50	0.10 (0.45)	0.89 (0.86–0.91)	21.0	0.02 (0.57)	0.81 (0.77–0.85)	27.6
8	20	0.06 (0.25)	0.94 (0.92–0.95)	19.0	0.04 (0.31)	0.89 (0.86–0.91)	24.5
8	30	0.06 (0.30)	0.92 (0.90–0.94)	19.0	0.03 (0.33)	0.89 (0.87–0.91)	21.5
8	40	0.08 (0.35)	0.93 (0.90–0.94)	19.1	0.05 (0.46)	0.84 (0.80–0.87)	25.8
8	50	0.07 (0.31)	0.94 (0.92–0.95)	15.5	0.05 (0.44)	0.85 (0.81–0.88)	22.7
10	20	0.05 (0.24)	0.95 (0.93–0.96)	19.2	0.01 (0.32)	0.90 (0.87–0.92)	26.1
10	30	0.07 (0.34)	0.93 (0.91–0.95)	20.2	0.04 (0.46)	0.87 (0.84–0.89)	28.7
10	40	0.10 (0.40)	0.92 (0.90–0.94)	21.1	0.04 (0.55)	0.84 (0.80–0.87)	30.0
10	50	0.10 (0.38)	0.92 (0.89–0.93)	19.0	0.12 (0.70)	0.84 (0.80–0.87)	35.9

*SD* standard deviation, *Diff* difference, *ICC* intraclass correlation coefficient, *CoV* coefficient of variation, *LV* left ventricular, *GLS* global longitudinal strain, *SRs *systolic strain rate (n = 336 data points)

#### Temporal resolution

Temporal resolution impacted neither intra- nor inter-observer reproducibility of LV strain values, including evaluations in segments with regional wall motion abnormalities only. However, increased temporal resolution was associated with better reproducibility of RV GLS, especially for inter-observer variability (CoV comparing 20 to 50 frames/cardiac cycle at 5 mm 17.3% vs 11.7%, 8 mm 14.2% vs 8.6% and at 10 mm 19.1% vs 12.8%).

#### Spatial resolution

Variation of spatial resolution between high, standard and low settings was not associated with decreased reproducibility of strain and SR values.

## Discussion

Temporal but not spatial resolution impacts CMR-FT absolute strain and SR values with higher temporal resolution being associated with increased strain and SR values. To obtain consistent numerically similar strain and SR values, temporal resolutions of above 30 (strain) and 50 frames/cardiac cycle (SR) are required to allow quantifying accurate peak systolic strain and SR. It is important to note that strain and SR acquired at lower temporal resolution will have lower peak systolic strain and SR which needs to be considered during clinical examinations. Within investigated limits of temporal and spatial resolution, reproducibility of LV strain and SR is not negatively affected by lower resolution settings. Consequently, when assessing these parameters clinically, minimum imaging requirements should be adhered to, to obtain reliable results for adequate diagnostic and prognostic assessment of patients with various cardiac pathologies.

### Technical considerations

#### Temporal resolution

Temporal resolution had the highest impact on strain and SR values assessed by FT post-processing. In line with data coming from STE [27] an increase in frame rate/temporal resolution was associated with an increase of absolute strain values.

One reason may be the CMR technique itself. First, CMR cine images of a cardiac cycle are retrospectively reconstructed from ECG-gated data of several cardiac cycles. Low temporal resolution may aggravate loss of information on minor differences in cardiac mechanics which may then not be adequately represented in the reconstructed cine image. Second, computation and extrapolation of strain curves from cine images with lower temporal resolution may result in loss of peak strain.

Another reason could be attributed to the FT technique. CMR-FT is in principle based on optical flow and aims to follow image patterns from frame to frame thoughout the cardiac cycle. The displacement is usually tracked between two consecutive images by identifying similar image patterns within an interrogation window [17, 26, 31]. A lower temporal resolution results in larger distances covered by the features from one image to the following one and thus requires an enlarged interrogation window to ensure that the patterns are not displaced beyond the limits of the interrogation window. Because pattern similarities are averaged over larger areas, accuracy may be decreased [26]. Furthermore, feature patterns in images may be less comparable after a longer period of time and larger distance covered, called image de-correlation [32]. In return, higher temporal resolution may allow for smaller interrogation windows resulting in higher accuracy. However, bearing also in mind that a too small search window may be unsuitable for pattern recognition as well [33]. This is in line with phantom work showing that a certain threshold of temporal resolution should be ensured whilst further increases may not automatically result in higher accuracy [34]. FT post-processing deformation assessment is based on estimations of displacements which are averaged for final values. On the one side this increases reproducibility and indeed, reproducibility was independent of temporal resolution. On the other side, it may also mean further loss of information in addition to temporal resolution.

Importantly, measurements such as velocities and SRs are computed as differentials of displacements/strain and thus are subject to higher inaccuracy compared to the latter [26]. In this case, temporal resolution gains further importance. Our data confirms strain rates to be most prone to changes in temporal resolution, with deviations in SR values amount up to 45% comparing the lowest to the highest temporal resolution.

#### Spatial resolution

Spatial resolution itself did neither impact absolute strain and SR values nor their reproducibility. When it comes to higher temporal resolutions, one needs to keep in mind that shorter displacements must also be detectable within the respective spatial resolution. Thus high temporal resolution requires higher spatial resolution [35]. Interestingly, the lowest spatial resolution was associated with the highest absolute increase in LV and RV GLS compared between increasing temporal resolutions. The longitudinal displacement of the atrio-ventricular junction is the motion with the largest distance covered during the cardiac cycle and distinctly contributes to overall LV/RV GLS. It can be speculated that higher spatial resolutions allow more detailed assessments and thus reduce variation caused by temporal resolution. Nevertheless, within investigated limits of temporal and spatial resolutions, a low spatial resolution sufficiently enabled strain and SR assessments with a recommended temporal resolution of 30 frames/cardiac cycle and beyond. Whether or not higher spatial resolutions are needed for temporal resolutions beyond 50 frames/cardiac cycle and allow for enhanced accuracy remains to be elucidated. However, considering clinical feasibility, temporal resolutions beyond 50 frames/cardiac cycle alongside with high spatial resolutions seem to be limited for research purposes.

### Clinical considerations

Considering normal LV GLS values of − 20% with a 95% CI of − 19.3 to − 20.9 [36], a deviation of 1.8% comparing the lowest and the highest temporal resolution is close to 10% in relative change of absolute strain values. This could be considered clinically relevant. Nevertheless, variability caused by temporal resolution attenuated beyond 30 frames/cardiac cycle, which is in line with data coming from STE [27]. This underlines that consistent strain assessment is achieved at 30 frames/cardiac cycle for clinical imaging. Importantly, LV strain evaluation has recently been included within the guidelines for cardiac functional assessment in case of preserved LVEF such as in HFpEF [8]. Strain rate imaging expands comprehensive cardiac functional evaluations with the possibility of velocity assessments as established in echocardiography [8]. For strain rate values a continuous increase of absolute values assessed by FT is observed with increasing temporal resolution. Hence, if one aims to image velocities or acceleration, temporal resolution gains further importance and a threshold of 30 frames/cardiac cycle may not be sufficient for reliable assessment, especially when it comes to ECG-gated reconstructed CMR images. To date, introduction of clinical deformation imaging has not been fully achieved since the different available techniques are not completely interchangeable [31] including deviations introduced by different vendors within a given approach [22, 28]. However, data also indicates high correlation of CMR-derived deformation imaging, pointing towards valid measurements by all different approaches, which however require specific reference values [37]. Lastly, in opposition to previous studies [20], the current paper now with the evolution of the underlying software algorithms indicates excellent CMR-FT reproducibility also on the segmental level, which makes this technology applicable to a larger disease spectrum including regional myocardial disease such as coronary disease. However, further harmonisation between vendors and/or specific reference values are required to allow full clinical implementation of this promising technology [11, 13].

## Limitations

The conclusions made are derived from CMR-FT data using a widely used commercially available software algorithm without a STE or a CMR tagging reference. However, the FT software used has previously been extensively validated. The findings made in this work apply to the specific software utilized. Since competitive software solutions of different vendors are based on non-disclosed and likely different algorithms the findings made in this work may not entirely apply to these solutions. Nonetheless sufficient inter-vendor reproducibility has been demonstrated for CMR-FT [38]. An impact of temporal resolution has been demonstrated on strain rate measurements, however systolic but not diastolic SR is reported. In contrast to STE, CMT-FT lacks animal validation work employing the reference standard for deformation imaging validation, sonomicrometry. However CMR-FT has been validated in phantom work and against harmonic phase imaging (tagging) [34] and strain encoding imaging (SENC) [38]. Healthy subjects and patients were all studied using a clinical 1.5 T CMR scanner. Nevertheless, differences are not anticipated since previous data suggests similar reproducibility of CMR-FT at 1.5 and 3 T [39].

## Conclusion

Temporal but not spatial resolution is an important source of varying strain and SR values in health and heart disease. Increasing temporal resolution is associated with increasing absolute strain and SR values. Strain assessments require lower temporal resolution than SR measurements. At 30 frames/cardiac cycle and beyond no significant changes in absolute values were observed for strain but for SR measurements. Within investigated limits, reproducibility is unaffected by spatial as well as temporal resolutions and best for LV GCS and GLS. Standardisation and official recommendations are required to allow correct deformation assessments in routine clinical practice, which should be based on further evidence based on future prospective large clinical research studies.

## Supplementary Information

**Additional file 1.** Additional data on reproducibility (Table S1–S5 and Figure S2–S13) as well as an examplary heart failure patient.

## Data Availability

Regarding data availability, we confirm that all relevant data are within the paper and all data underlying the findings are fully available without restriction and can be accessed at the University Medical Centre Goettingen by researchers who meet the criteria for access to confidential data.

## Abbreviations

2C: Two chamber
4C: Four-chamber
bSSFP: Balanced steady state free precession
CoV: Coefficient of variation
CMR: Cardiovascular magnetic resonance
ECG: Electrocardiogram
ED: End-diastole
ES: End-systole
FT: Feature tracking
ε: Strain
GCS: Global circumferential strain
GLS: Global longitudinal strain
HF: Heart failure
HFpEF: Heart failure with preserved ejection fraction
HFrEF: Heart failure with reduced ejectrion fraction
ICC: Intra-class correlation coefficient
LV: Left ventricle/left ventricular
LVEF: Left ventricular ejection fraction
MD: Mean difference
RV: Right ventricle/right ventricular
SD: Standard deviation
SR: Strain rate
STE: Speckle tracking echocardiography

## References

[1] Bergenzaun L, Ohlin H, Gudmundsson P, Willenheimer R, Chew MS (2013). Mitral annular plane systolic excursion (MAPSE) in shock: a valuable echocardiographic parameter in intensive care patients. Cardiovasc Ultrasound.

[2] Stanton T, Leano R, Marwick TH (2009). Prediction of all-cause mortality from global longitudinal speckle strain: comparison with ejection fraction and wall motion scoring. Circ Cardiovasc Imaging.

[3] Neizel M, Korosoglou G, Lossnitzer D, Kühl H, Hoffmann R, Ocklenburg C (2010). Impact of systolic and diastolic deformation indexes assessed by strain-encoded imaging to predict persistent severe myocardial dysfunction in patients after acute myocardial infarction at follow-up. J Am Coll Cardiol.

[4] Mordi I, Bezerra H, Carrick D, Tzemos N (2015). The combined incremental prognostic value of LVEF, late gadolinium enhancement, and global circumferential strain assessed by CMR. JACC Cardiovasc Imaging.

[5] Mangion K, Carrick D, Carberry J, Mahrous A, McComb C, Oldroyd KG (2018). Circumferential strain predicts major adverse cardiovascular events following an acute ST-segment-elevation myocardial infarction. Radiology..

[6] Park JJ, Park J-B, Park J-H, Cho G-Y (2018). Global longitudinal strain to predict mortality in patients with acute heart failure. J Am Coll Cardiol.

[7] Russo C, Jin Z, Elkind MSV, Rundek T, Homma S, Sacco RL, Di Tullio MR (2014). Prevalence and prognostic value of subclinical left ventricular systolic dysfunction by global longitudinal strain in a community-based cohort. Eur J Heart Fail.

[8] Nagueh SF, Smiseth OA, Appleton CP, Byrd BF, Dokainish H, Edvardsen T (2016). Recommendations for the evaluation of left ventricular diastolic function by echocardiography: an update from the american society of echocardiography and the european association of cardiovascular imaging. Eur Heart J Cardiovasc Imaging.

[9] Buss SJ, Breuninger K, Lehrke S, Voss A, Galuschky C, Lossnitzer D (2015). Assessment of myocardial deformation with cardiac magnetic resonance strain imaging improves risk stratification in patients with dilated cardiomyopathy. Eur Heart J Cardiovasc Imaging.

[10] Romano S, Judd RM, Kim RJ, Kim HW, Klem I, Heitner JF (2018). Feature-tracking global longitudinal strain predicts death in a multicenter population of patients with ischemic and nonischemic dilated cardiomyopathy incremental to ejection fraction and late gadolinium enhancement. JACC Cardiovasc Imaging.

[11] Eitel I, Stiermaier T, Lange T, Rommel K-P, Koschalka A, Kowallick JT (2018). Cardiac magnetic resonance myocardial feature tracking for optimized prediction of cardiovascular events following myocardial infarction. JACC Cardiovasc Imaging.

[12] Ersbøll M, Valeur N, Mogensen UM, Andersen MJ, Møller JE, Velazquez EJ (2013). Prediction of all-cause mortality and heart failure admissions from global left ventricular longitudinal strain in patients with acute myocardial infarction and preserved left ventricular ejection fraction. J Am Coll Cardiol.

[13] Biering-Sørensen T, Jensen JS, Pedersen SH, Galatius S, Fritz-Hansen T, Bech J (2016). Regional Longitudinal Myocardial Deformation Provides Incremental Prognostic Information in Patients with ST-Segment Elevation Myocardial Infarction. PLoS ONE.

[14] Pennell DJ (2010). Cardiovascular magnetic resonance. Circulation.

[15] Cao JJ, Ngai N, Duncanson L, Cheng J, Gliganic K, Chen Q (2018). A comparison of both DENSE and feature tracking techniques with tagging for the cardiovascular magnetic resonance assessment of myocardial strain. J Cardiovasc Magn Reson.

[16] Giusca S, Korosoglou G, Zieschang V, Stoiber L, Schnackenburg B, Stehning C (2018). Reproducibility study on myocardial strain assessment using fast-SENC cardiac magnetic resonance imaging. Sci Rep.

[17] Schuster A, Hor KN, Kowallick JT, Beerbaum P, Kutty S (2016). Cardiovascular magnetic resonance myocardial feature tracking: concepts and clinical applications. Circ Cardiovasc Imaging.

[18] Scatteia A, Baritussio A, Bucciarelli-Ducci C (2017). Strain imaging using cardiac magnetic resonance. Heart Fail Rev.

[19] Kowallick JT, Morton G, Lamata P, Jogiya R, Kutty S, Lotz J (2016). Inter-study reproducibility of left ventricular torsion and torsion rate quantification using MR myocardial feature tracking. J Magn Reson Imaging.

[20] Morton G, Schuster A, Jogiya R, Kutty S, Beerbaum P, Nagel E (2012). Inter-study reproducibility of cardiovascular magnetic resonance myocardial feature tracking. J Cardiovasc Magn Reson.

[21] Schuster A, Stahnke V-C, Unterberg-Buchwald C, Kowallick JT, Lamata P, Steinmetz M (2015). Cardiovascular magnetic resonance feature-tracking assessment of myocardial mechanics: Intervendor agreement and considerations regarding reproducibility. Clin Radiol.

[22] Gertz RJ, Lange T, Kowallick JT, Backhaus SJ, Steinmetz M, Staab W (2018). Inter-vendor reproducibility of left and right ventricular cardiovascular magnetic resonance myocardial feature-tracking. PLoS ONE.

[23] Schuster A, Kutty S, Padiyath A, Parish V, Gribben P, Danford DA (2011). Cardiovascular magnetic resonance myocardial feature tracking detects quantitative wall motion during dobutamine stress. J Cardiovasc Magn Reson.

[24] Schuster A, Paul M, Bettencourt N, Morton G, Chiribiri A, Ishida M (2013). Cardiovascular magnetic resonance myocardial feature tracking for quantitative viability assessment in ischemic cardiomyopathy. Int J Cardiol.

[25] Orwat S, Kempny A, Diller G-P, Bauerschmitz P, Bunck AC, Maintz D, et al. Cardiac magnetic resonance feature tracking: a novel method to assess myocardial strain. Comparison with echocardiographic speckle tracking in healthy volunteers and in patients with left ventricular hypertrophy. Kardiol Pol. 2014;72:363–71. doi:10.5603/KP.a2013.0319. 10.5603/KP.a2013.0319 24293146

[26] Pedrizzetti G, Claus P, Kilner PJ, Nagel E (2016). Principles of cardiovascular magnetic resonance feature tracking and echocardiographic speckle tracking for informed clinical use. J Cardiovasc Magn Reson.

[27] Rösner A, Barbosa D, Aarsæther E, Kjønås D, Schirmer H, d'Hooge J (2015). The influence of frame rate on two-dimensional speckle-tracking strain measurements: a study on silico-simulated models and images recorded in patients. Eur Heart J Cardiovasc Imaging.

[28] Backhaus SJ, Metschies G, Billing M, Kowallick JT, Gertz RJ, Lapinskas T (2019). Cardiovascular magnetic resonance imaging feature tracking: Impact of training on observer performance and reproducibility. PLoS ONE.

[29] Cerqueira MD, Weissman NJ, Dilsizian V, Jacobs AK, Kaul S, Laskey WK (2002). Standardized myocardial segmentation and nomenclature for tomographic imaging of the heart. Circulation.

[30] Bland M, Altman D (1986). Statistical Methods for assessing agreement between two methods of clinical measurement. The Lancet.

[31] Amzulescu MS, de Craene M, Langet H, Pasquet A, Vancraeynest D, Pouleur AC (2019). Myocardial strain imaging: review of general principles, validation, and sources of discrepancies. Eur Heart J Cardiovasc Imaging.

[32] Liang T, Yung L, Yu W (2013). On feature motion decorrelation in ultrasound speckle tracking. IEEE Trans Med Imaging.

[33] Barron JL, Fleet DJ, Beauchemin SS (1994). Performance of optical flow techniques. Int J Comput Vision.

[34] Hor KN, Baumann R, Pedrizzetti G, Tonti G, Gottliebson WM, Taylor M (2011). Magnetic resonance derived myocardial strain assessment using feature tracking. J Vis Exp.

[35] Adrian RJ (1991). Particle-Imaging Techniques for Experimental Fluid Mechanics. Annu Rev Fluid Mech.

[36] Vo HQ, Marwick TH, Negishi K (2018). MRI-derived myocardial strain measures in normal subjects. JACC Cardiovasc Imaging.

[37] Bucius P, Erley J, Tanacli R, Zieschang V, Giusca S, Korosoglou G (2019). Comparison of feature tracking, fast-SENC, and myocardial tagging for global and segmental left ventricular strain. ESC Heart Fail.

[38] Backhaus SJ, Metschies G, Zieschang V, Erley J, Mahsa Zamani S, Kowallick JT (2021). Head-to-head comparison of cardiovascular MR feature tracking cine versus acquisition-based deformation strain imaging using myocardial tagging and strain encoding. Magn Reson Med.

[39] Schuster A, Morton G, Hussain ST, Jogiya R, Kutty S, Asrress KN (2013). The intra-observer reproducibility of cardiovascular magnetic resonance myocardial feature tracking strain assessment is independent of field strength. Eur J Radiol.

