# Supplementary material for: Defining the optimal temporal and spatial resolution for cardiovascular magnetic resonance imaging feature tracking
Source: J Cardiovasc Magn Reson. 2021 May 17;23:60. doi: 10.1186/s12968-021-00740-5 (PMC8127257; doi:10.1186/s12968-021-00740-5)
Supplement: Supplementary file 1 — Additional file 1. Additional data on reproducibility (Table S1–S5 and Figure S2–S13) as well as an examplary heart failure patient. [file 12968_2021_740_MOESM1_ESM.docx]

**Reproducibility Analyses**

| Resolution | | Intra-Observer |  |  | Inter-Observer |  |  |
| --- | --- | --- | --- | --- | --- | --- | --- |
| Spatial (mm) | Temporal (frames/cycle) | Mean Difference  (SD of the Diff.) | ICC (95% CI) | CoV (%) | Mean Difference  (SD of the Diff.) | ICC (95% CI) | CoV (%) |
| 5 | 20 | 1.31 (3.22) | 0.70 (0.29-0.88) | 13.4 | 0.73 (4.11) | 0.54 (0.00-0.81) | 17.3 |
| 5 | 30 | 1.15 (3.01) | 0.73 (0.35-0.89) | 12.1 | 1.29 (3.77) | 0.66 (0.21-0.86) | 15.1 |
| 5 | 40 | 0.81 (2.62) | 0.77 (0.43-0.90) | 10.5 | 0.89 (3.77) | 0.65 (0.15-0.86) | 15.1 |
| 5 | 50 | 0.80 (2.47) | 0.80 (0.51-0.92) | 10.0 | 0.79 (2.92) | 0.78 (0.47-0.91) | 11.7 |
| 8 | 20 | 0.85 (2.79) | 0.79 (0.47-0.91) | 11.5 | 0.38 (3.40) | 0.73 (0.34-0.89) | 14.2 |
| 8 | 30 | 2.04 (2.36) | 0.79 (0.21-0.93) | 9.5 | 1.03 (3.00) | 0.82 (0.57-0.93) | 12.3 |
| 8 | 40 | 1.06 (2.37) | 0.84 (0.60-0.94) | 9.4 | 0.52 (3.37) | 0.78 (0.45-0.91) | 13.5 |
| 8 | 50 | 0.77 (3.08) | 0.77 (0.43-0.90) | 12.2 | 0.52 (2.16) | 0.91 (0.78-0.96) | 8.6 |
| 10 | 20 | 0.78 (3.99) | 0.70 (0.28-0.88) | 17.1 | 0.18 (4.38) | 0.70 (0.26-0.88) | 19.1 |
| 10 | 30 | 0.45 (2.86) | 0.73 (0.34-0.89) | 11.7 | 0.70 (2.95) | 0.76 (0.42-0.90) | 12.2 |
| 10 | 40 | 0.54 (2.96) | 0.79 (0.50-0.92) | 11.9 | 0.45 (2.95) | 0.83 (0.58-0.93) | 12.1 |
| 10 | 50 | 0.92 (2.48) | 0.79 (0.49-0.92) | 9.8 | 0.60 (3.14) | 0.74 (0.36-0.90) | 12.8 |
| **Table S1 RV GLS intra- and inter-observer reproducibility** SD: standard deviation. Diff: Difference. ICC: intraclass correlation coefficient. CoV: coefficient of variation. RV: right ventricular. GLS: global longitudinal strain. (n= 21 data points). | | | | | | | |

| Resolution | | Intra-Observer |  |  | Inter-Observer |  |  |
| --- | --- | --- | --- | --- | --- | --- | --- |
| Spatial (mm) | Temporal (frames/cycle) | Mean Difference  (SD of the Diff.) | ICC (95% CI) | CoV (%) | Mean Difference  (SD of the Diff.) | ICC (95% CI) | CoV (%) |
| 5 | 20 | 0.87 (3.24) | 0.96 (0.91-0.98) | 19.0 | 0.97 (4.60) | 0.91 (0.82-0.95) | 27.1 |
| 5 | 30 | 0.09 (2.81) | 0.97 (0.94-0.98) | 15.6 | 0.37 (4.17) | 0.93 (0.86-0.96) | 22.9 |
| 5 | 40 | 0.03 (2.80) | 0.97 (0.94-0.98) | 15.4 | 0.57 (3.51) | 0.95 (0.90-0.97) | 19.0 |
| 5 | 50 | 1.07 (3.34) | 0.96 (0.92-0.98) | 18.8 | 0.83 (4.69) | 0.93 (0.86-0.96) | 25.1 |
| 8 | 20 | 0.05 (2.55) | 0.97 (0.95-0.99) | 14.0 | 1.34 (4.86) | 0.89 (0.79-0.95) | 27.6 |
| 8 | 30 | 0.01 (2.78) | 0.97 (0.94-0.98) | 16.5 | 0.16 (4.17) | 0.92 (0.85-0.96) | 24.8 |
| 8 | 40 | 0.30 (2.22) | 0.98 (0.97-0.99) | 12.4 | 0.59 (4.77) | 0.91 (0.82-0.95) | 27.0 |
| 8 | 50 | 0.35 (2.12) | 0.99 (0.97-0.99) | 11.2 | 0.37 (4.52) | 0.92 (0.85-0.96) | 24.0 |
| 10 | 20 | 0.21 (3.81) | 0.95 (0.90-0.97) | 22.9 | 1.34 (5.15) | 0.86 (0.73-0.93) | 32.0 |
| 10 | 30 | 0.87 (2.27) | 0.99 (0.97-0.99) | 12.3 | 1.08 (4.83) | 0.93 (0.87-0.97) | 26.2 |
| 10 | 40 | 0.75 (2.96) | 0.97 (0.95-0.99) | 15.7 | 0.40 (4.60) | 0.93 (0.87-0.97) | 24.2 |
| 10 | 50 | 0.17 (2.93) | 0.97 (0.95-0.99) | 15.9 | 0.06 (5.46) | 0.89 (0.79-0.94) | 29.6 |
| **Table S2 LV GLS intra- and inter-observer reproducibility – abnormal wall motion** SD: standard deviation. Diff: Difference. ICC: intraclass correlation coefficient. CoV: coefficient of variation. LV: left ventricular. GLS: global longitudinal strain. (n=35 data points). | | | | | | | |

| Resolution | | Intra-Observer |  |  | Inter-Observer |  |  |
| --- | --- | --- | --- | --- | --- | --- | --- |
| Spatial (mm) | Temporal (frames/cycle) | Mean Difference  (SD of the Diff.) | ICC (95% CI) | CoV (%) | Mean Difference  (SD of the Diff.) | ICC (95% CI) | CoV (%) |
| 5 | 20 | 0.03 (0.13) | 0.96 (0.92-0.98) | 17.2 | 0.05 (0.18) | 0.92 (0.84-0.96) | 24.9 |
| 5 | 30 | 0.03 (0.11) | 0.98 (0.95-0.99) | 11.9 | 0.04 (0.22) | 0.90 (0.81-0.95) | 23.6 |
| 5 | 40 | 0.01 (0.23) | 0.91 (0.82-0.95) | 21.9 | 0.00 (0.20) | 0.94 (0.88-0.97) | 19.0 |
| 5 | 50 | 0.00 (0.14) | 0.97 (0.95-0.99) | 12.1 | 0.05 (0.19) | 0.95 (0.90-0.97) | 16.2 |
| 8 | 20 | 0.01 (0.11) | 0.98 (0.96-0.99) | 13.4 | 0.02 (0.20) | 0.92 (0.85-0.96) | 25.4 |
| 8 | 30 | 0.01 (0.13) | 0.97 (0.94-0.98) | 14.5 | 0.02 (0.18) | 0.94 (0.87-0.97) | 19.3 |
| 8 | 40 | 0.03 (0.11) | 0.98 (0.96-0.99) | 10.4 | 0.03 (0.22) | 0.92 (0.85-0.96) | 21.0 |
| 8 | 50 | 0.05 (0.15) | 0.98 (0.97-0.99) | 11.1 | 0.07 (0.40) | 0.84 (0.69-0.92) | 30.4 |
| 10 | 20 | 0.00 (0.16) | 0.95 (0.90-0.97) | 21.3 | 0.06 (0.23) | 0.82 (0.66-0.91) | 32.3 |
| 10 | 30 | 0.00 (0.16) | 0.98 (0.97-0.99) | 15.0 | 0.04 (0.25) | 0.95 (0.91-0.98) | 23,9 |
| 10 | 40 | 0.01 (0.14) | 0.97 (0.94-0.98) | 13.3 | 0.00 (0.27) | 0.88 (0.75-0.94) | 26.3 |
| 10 | 50 | 0.03 (0.19) | 0.95 (0.91-0.98) | 15.3 | 0.00 (0.33) | 0.86 (0.72-0.93) | 26.2 |
| **Table S3 LV GLS SR intra- and inter-observer reproducibility – abnormal wall motion** SD: standard deviation. Diff: Difference. ICC: intraclass correlation coefficient. CoV: coefficient of variation. LV: left ventricular. SRs: systolic strain rate. (n=35 data points). | | | | | | | |

| Resolution | | Intra-Observer |  |  | Inter-Observer |  |  |
| --- | --- | --- | --- | --- | --- | --- | --- |
| Spatial (mm) | Temporal (frames/cycle) | Mean Difference  (SD of the Diff.) | ICC (95% CI) | CoV (%) | Mean Difference  (SD of the Diff.) | ICC (95% CI) | CoV (%) |
| 5 | 20 | 0.29 (2.72) | 0.98 (0.96-0.99) | 13.3 | 1.00 (7.00) | 0.87 (0.73-0.93) | 33.2 |
| 5 | 30 | 0.50 (2.83) | 0.98 (0.97-0.99) | 12.4 | 0.32 (6.45) | 0.91 (0.83-0.96) | 28.2 |
| 5 | 40 | 0.02 (2.21) | 0.99 (0.98-0.99) | 9.1 | 0.08 (7.29) | 0.84 (0.67-0.92) | 29.9 |
| 5 | 50 | 0.39 (2.98) | 0.97 (0.95-0.99) | 12.6 | 0.22 (6.49) | 0.86 (0.73-0.93) | 27.4 |
| 8 | 20 | 0.27 (3.16) | 0.96 (0.92-0.98) | 14.4 | 0.53 (8.60) | 0.73 (0.45-0.86) | 40.0 |
| 8 | 30 | 0.10 (3.44) | 0.96 (0.91-0.98) | 15.4 | 0.42 (7.63) | 0.77 (0.53-0.88) | 33.9 |
| 8 | 40 | 0.02 (2.69) | 0.98 (0.95-0.99) | 11.6 | 0.08 (6.76) | 0.86 (0.71-0.93) | 29.1 |
| 8 | 50 | 0.21 (3.35) | 0.96 (0.92-0.98) | 13.9 | 0.05 (6.73) | 0.84 (0.68-0.92) | 28.0 |
| 10 | 20 | 0.83 (2.94) | 0.97 (0.94-0.99) | 14.1 | 1.51 (7.35) | 0.82 (0.64-0.91) | 34.6 |
| 10 | 30 | 0.42 (2.77) | 0.98 (0.96-0.99) | 11.9 | 0.66 (9.08) | 0.79 (0.58-0.89) | 39.1 |
| 10 | 40 | 0.76 (3.11) | 0.98 (0.95-0.99) | 13.3 | 0.61 (7.82) | 0.85 (0.71-0.93) | 33.4 |
| 10 | 50 | 0.25 (3.39) | 0.97 (0.93-0.98) | 14.4 | 0.31 (7.45) | 0.84 (0.68-0.92) | 31.7 |
| **Table S4 LV GCS intra- and inter-observer reproducibility – abnormal wall motion** SD: standard deviation. Diff: Difference. ICC: intraclass correlation coefficient. CoV: coefficient of variation. LV: left ventricular. GCS: global circumferential strain. (n=35 data points). | | | | | | | |

| Resolution | | Intra-Observer |  |  | Inter-Observer |  |  |
| --- | --- | --- | --- | --- | --- | --- | --- |
| Spatial (mm) | Temporal (frames/cycle) | Mean Difference  (SD of the Diff.) | ICC (95% CI) | CoV (%) | Mean Difference  (SD of the Diff.) | ICC (95% CI) | CoV (%) |
| 5 | 20 | 0.03 (0.11) | 0.99 (0.97-0.99) | 11.8 | 0.08 (0.27) | 0.92 (0.84-0.96) | 27.8 |
| 5 | 30 | 0.04 (0.17) | 0.98 (0.96-0.99) | 13.4 | 0.01 (0.31) | 0.94 (0.89-0.97) | 23.6 |
| 5 | 40 | 0.04 (0.20) | 0.98 (0.96-0.99) | 13.1 | 0.02 (0.37) | 0.92 (0.83-0.96) | 24.2 |
| 5 | 50 | 0.01 (0.26) | 0.97 (0.94-0.99) | 15.8 | 0.03 (0.45) | 0.89 (0.78-0.95) | 27.3 |
| 8 | 20 | 0.01 (0.17) | 0.96 (0.92-0.98) | 18.0 | 0.04 (0.30) | 0.86 (0.73-0.93) | 31.7 |
| 8 | 30 | 0.01 (0.18) | 0.97 (0.94-0.98) | 14.9 | 0.04 (0.35) | 0.86 (0.72-0.93) | 29.9 |
| 8 | 40 | 0.01 (0.18) | 0.97 (0.94-0.98) | 13.6 | 0.04 (0.33) | 0.90 (0.79-0.95) | 24.1 |
| 8 | 50 | 0.01 (0.23) | 0.96 (0.92-0.98) | 15.4 | 0.06 (0.37) | 0.89 (0.79-0.95) | 24.3 |
| 10 | 20 | 0.01 (0.10) | 0.99 (0.98-1.00) | 10.7 | 0.06 (0.26) | 0.92 (0.85-0.96) | 28.3 |
| 10 | 30 | 0.03 (0.20) | 0.97 (0.95-0.99) | 16.4 | 0.00 (0.41) | 0.89 (0.77-0.94) | 0.32 |
| 10 | 40 | 0.00 (0.20) | 0.97 (0.94-0.98) | 15.0 | 0.05 (0.33) | 0.92 (0.84-0.96) | 23.8 |
| 10 | 50 | 0.04 (0.26) | 0.95 (0.91-0.98) | 17.5 | 0.05 (0.28) | 0.95 (0.90-0.97) | 18.6 |
| **Table S5 LV GCS SR intra- and inter-observer reproducibility – abnormal wall motion** SD: standard deviation. Diff: Difference. ICC: intraclass correlation coefficient. CoV: coefficient of variation. LV: left ventricular. SRs: systolic strain rate. (n=35 data points). | | | | | | | |


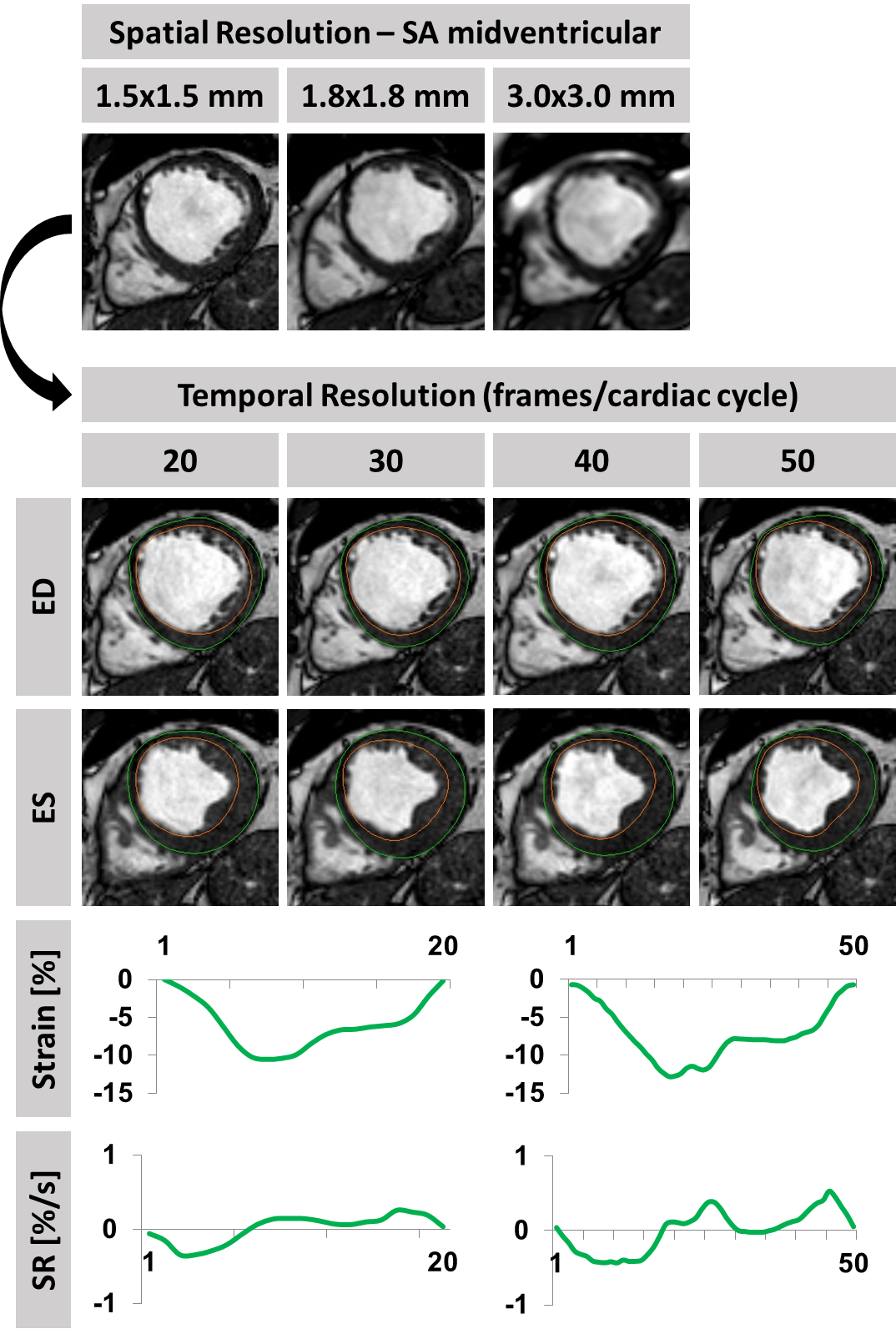


**Figure 1S – Strain and strain rate (SR) analyses with different spatial and temporal resolutions**

Example of a heart failure patient and end-diastolic midventricular short axis (SA) views using high (1.5x1.5 mm in plane and 5 mm through-plane), standard (1.8x1.8 mm in plane and 8mm through-plane) and low (3.0x3.0 mm in plane and 10 mm through-plane) spatial resolution. Below different temporal resolutions of 20, 30, 40 and 50 frames/cardiac cycle in high spatial resolution (1.5x1.5 mm in plane and 5 mm through-plane) with traced borders at end-diastole (ED) and end-systole (ES) are displayed. Strain and SR curves for GCS are exemplary shown for high spatial with either lowest (20) or highest (50) temporal resolution. Final strain values are based on endocardial strain only.


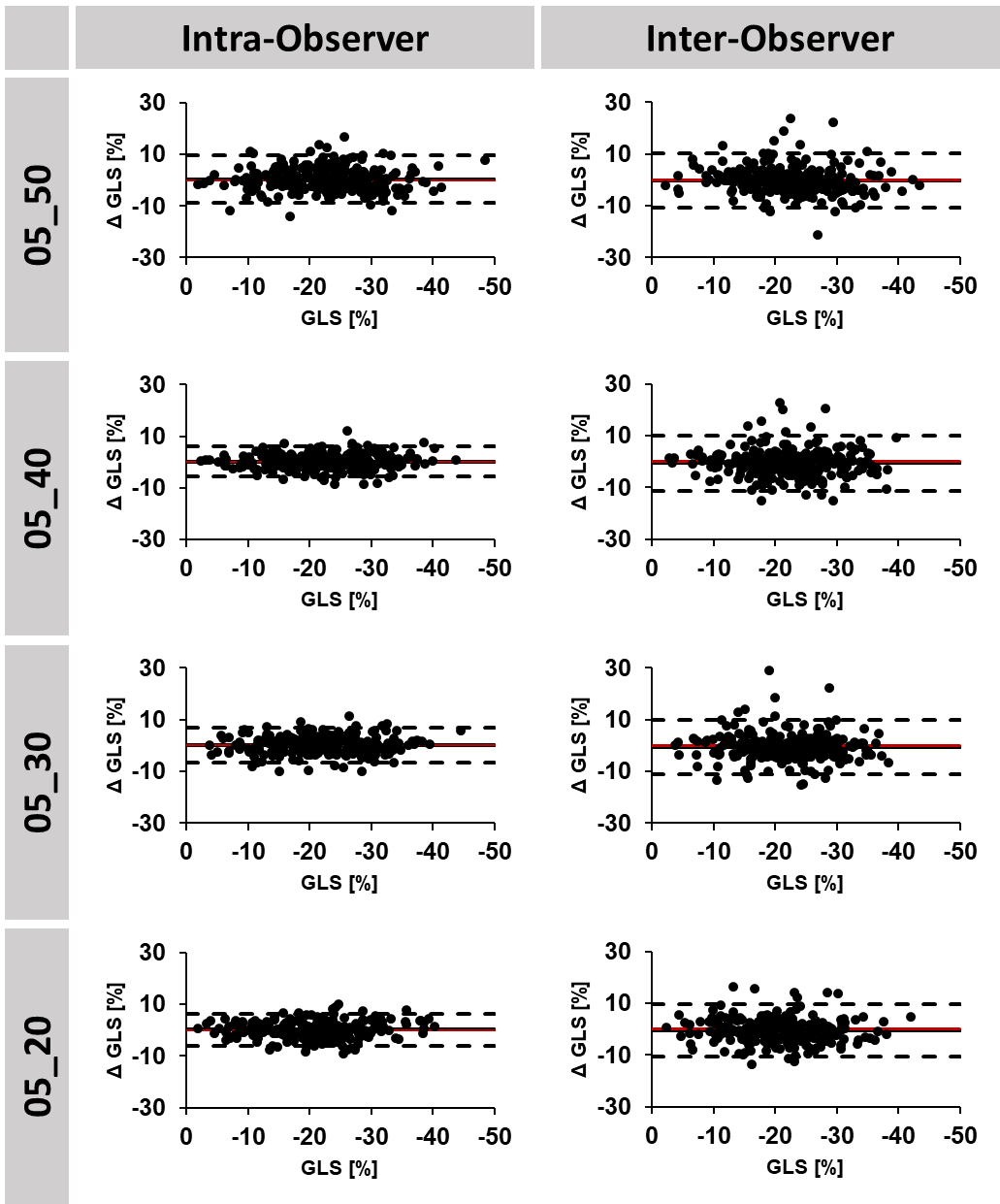


**Figure 2S Agreement of FT-GLS depending on Resolution (5 mm and 20-50 frames/cardiac cycle)**

Bland Altman plots are shown for intra- and inter-observer reproducibility of left ventricular global longitudinal strain (GLS) obtained by Feature-Tracking (FT) depending on spatial (1.5x1.5 mm in plane and 5 mm through-plane) and temporal (20-50 frames/cardiac cycle) resolution. Δ= difference for intra-observer (observer 1 first measurement – second measurement) or inter-observer (observer 1 first measurement – observer 2 measurement) measurements, respectively. Reference for 0 difference in red. (n=252 data points).


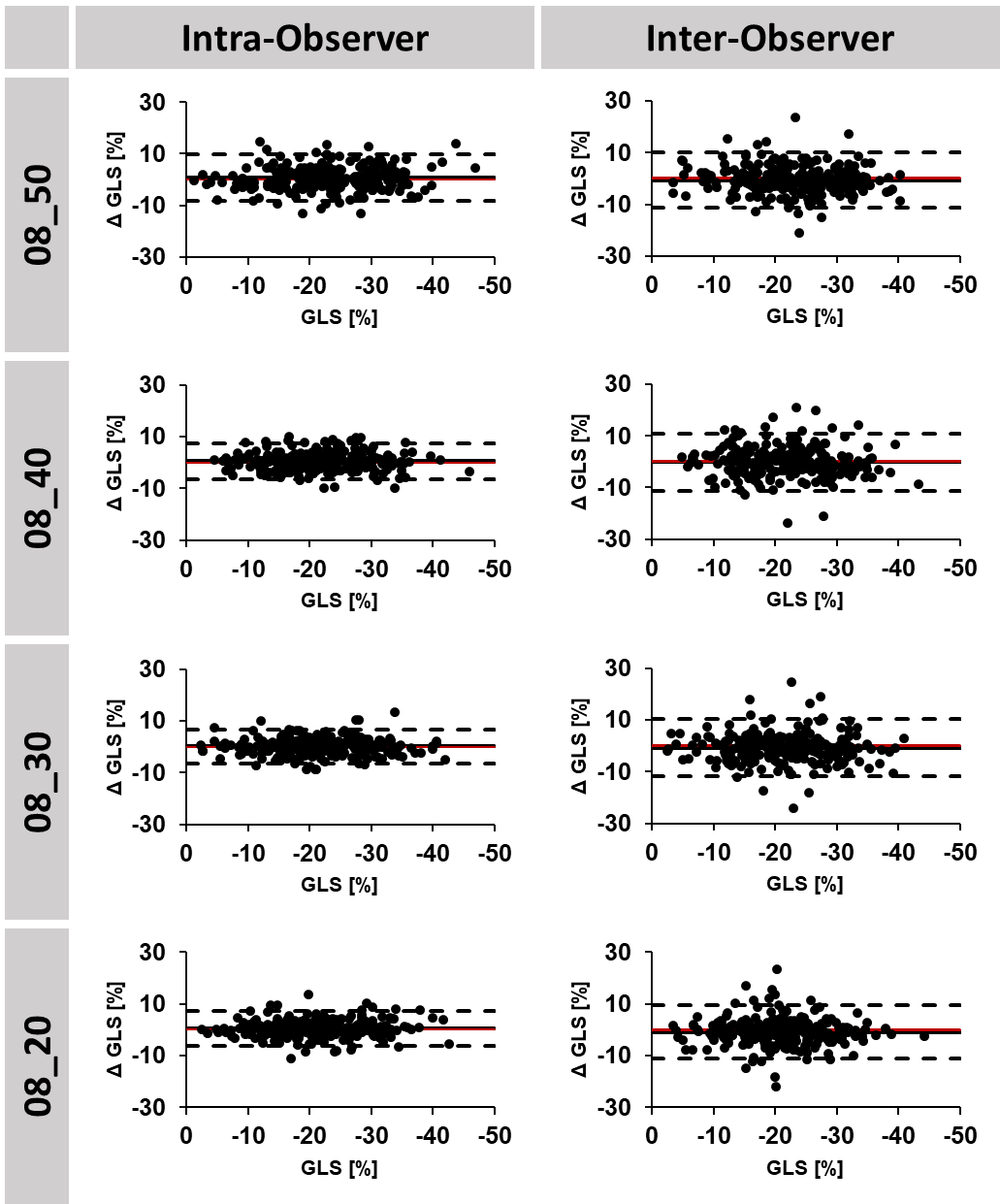


**Figure 3S Agreement of FT-GLS depending on Resolution (8 mm and 20-50 frames/cardiac cycle)**

Bland Altman plots are shown for intra- and inter-observer reproducibility of left ventricular global longitudinal strain (GLS) obtained by Feature-Tracking (FT) depending on spatial (1.8x1.8 mm in plane and 8 mm through-plane) and temporal (20-50 frames/cardiac cycle) resolution. Δ= difference for intra-observer (observer 1 first measurement – second measurement) or inter-observer (observer 1 first measurement – observer 2 measurement) measurements, respectively. Reference for 0 difference in red. (n=252 data points).


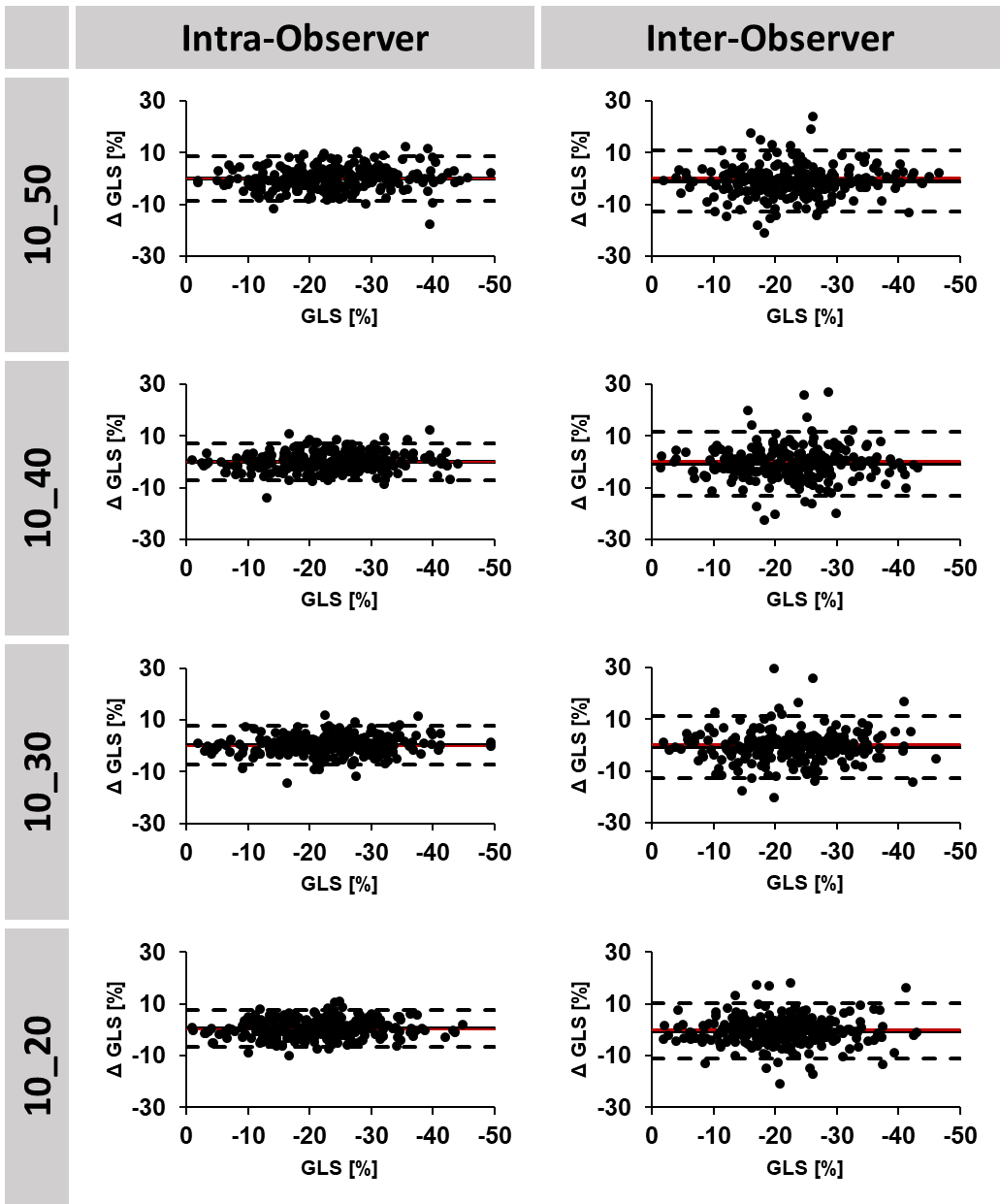


**Figure 4S Agreement of FT-GLS depending on Resolution (10 mm and 20-50 frames/cardiac cycle)**

Bland Altman plots are shown for intra- and inter-observer reproducibility of left ventricular global longitudinal strain (GLS) obtained by Feature-Tracking (FT) depending on spatial (3.0x3.0 mm in plane and 10 mm through-plane) and temporal (20-50 frames/cardiac cycle) resolution. Δ= difference for intra-observer (observer 1 first measurement – second measurement) or inter-observer (observer 1 first measurement – observer 2 measurement) measurements, respectively. Reference for 0 difference in red. (n=252 data points).


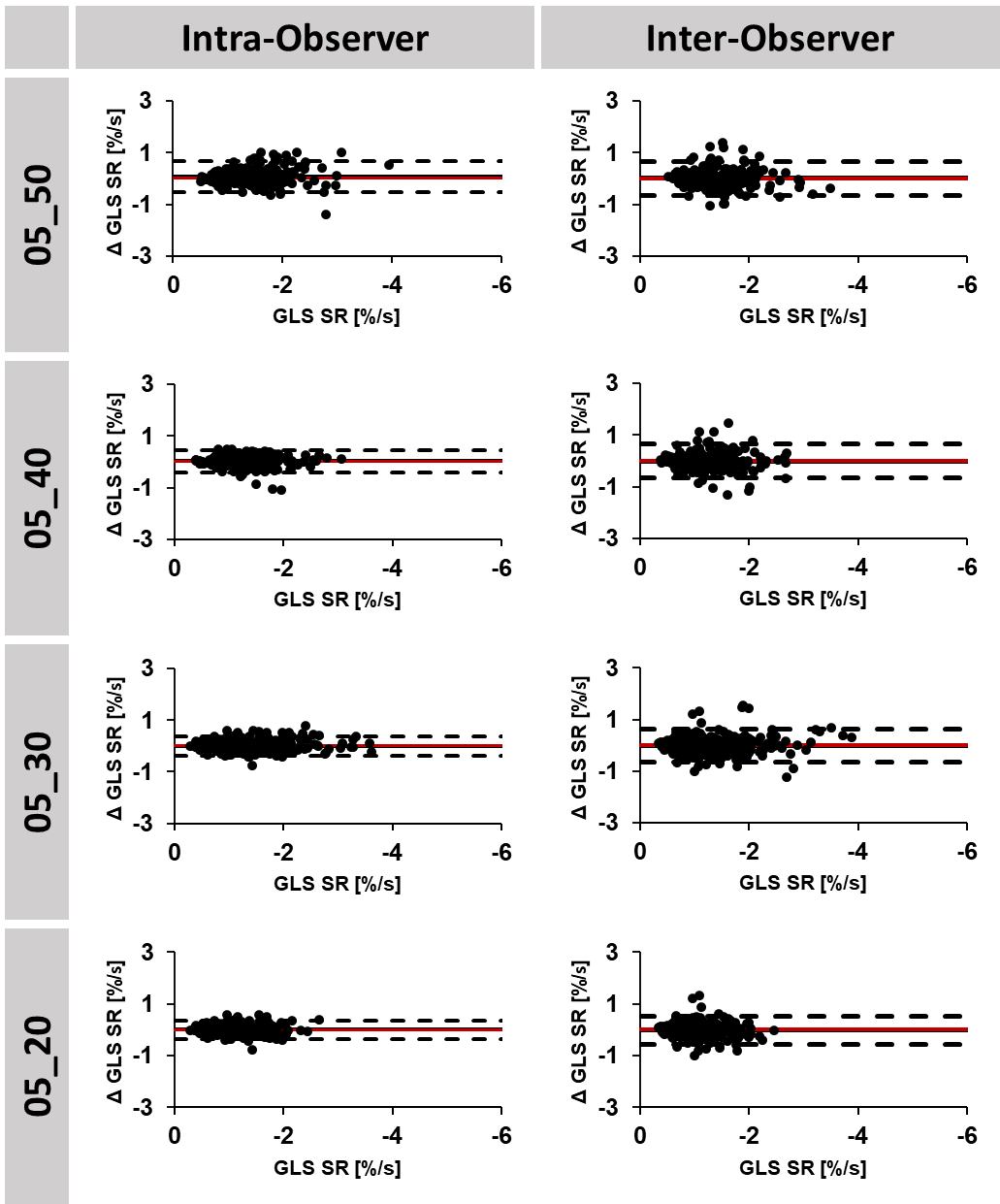


**Figure 5S Agreement of FT-GLS SR depending on Resolution (5 mm and 20-50 frames/cardiac cycle)**

Bland Altman plots are shown for intra- and inter-observer reproducibility of left ventricular global longitudinal strain rate (GLS SR) obtained by Feature-Tracking (FT) depending on spatial (1.5x1.5 mm in plane and 5 mm through-plane) and temporal (20-50 frames/cardiac cycle) resolution. Δ= difference for intra-observer (observer 1 first measurement – second measurement) or inter-observer (observer 1 first measurement – observer 2 measurement) measurements, respectively. Reference for 0 difference in red. (n=252 data points).


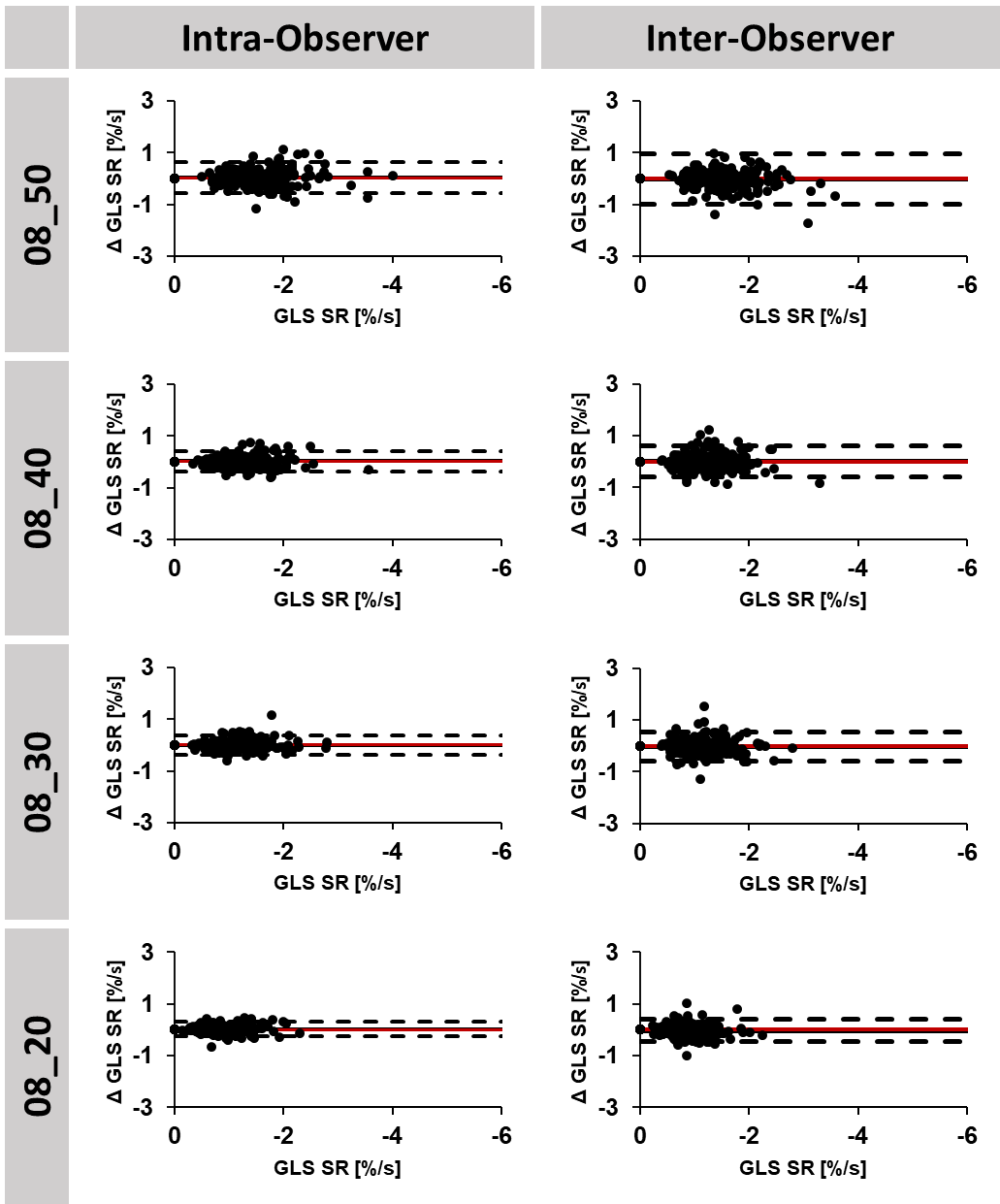


**Figure 6S Agreement of FT-GLS SR depending on Resolution (8 mm and 20-50 frames/cardiac cycle)**

Bland Altman plots are shown for intra- and inter-observer reproducibility of left ventricular global longitudinal strain rate (GLS SR) obtained by Feature-Tracking (FT) depending on spatial (1.8x1.8 mm in plane and 8 mm through-plane) and temporal (20-50 frames/cardiac cycle) resolution. Δ= difference for intra-observer (observer 1 first measurement – second measurement) or inter-observer (observer 1 first measurement – observer 2 measurement) measurements, respectively. Reference for 0 difference in red. (n=252 data points).


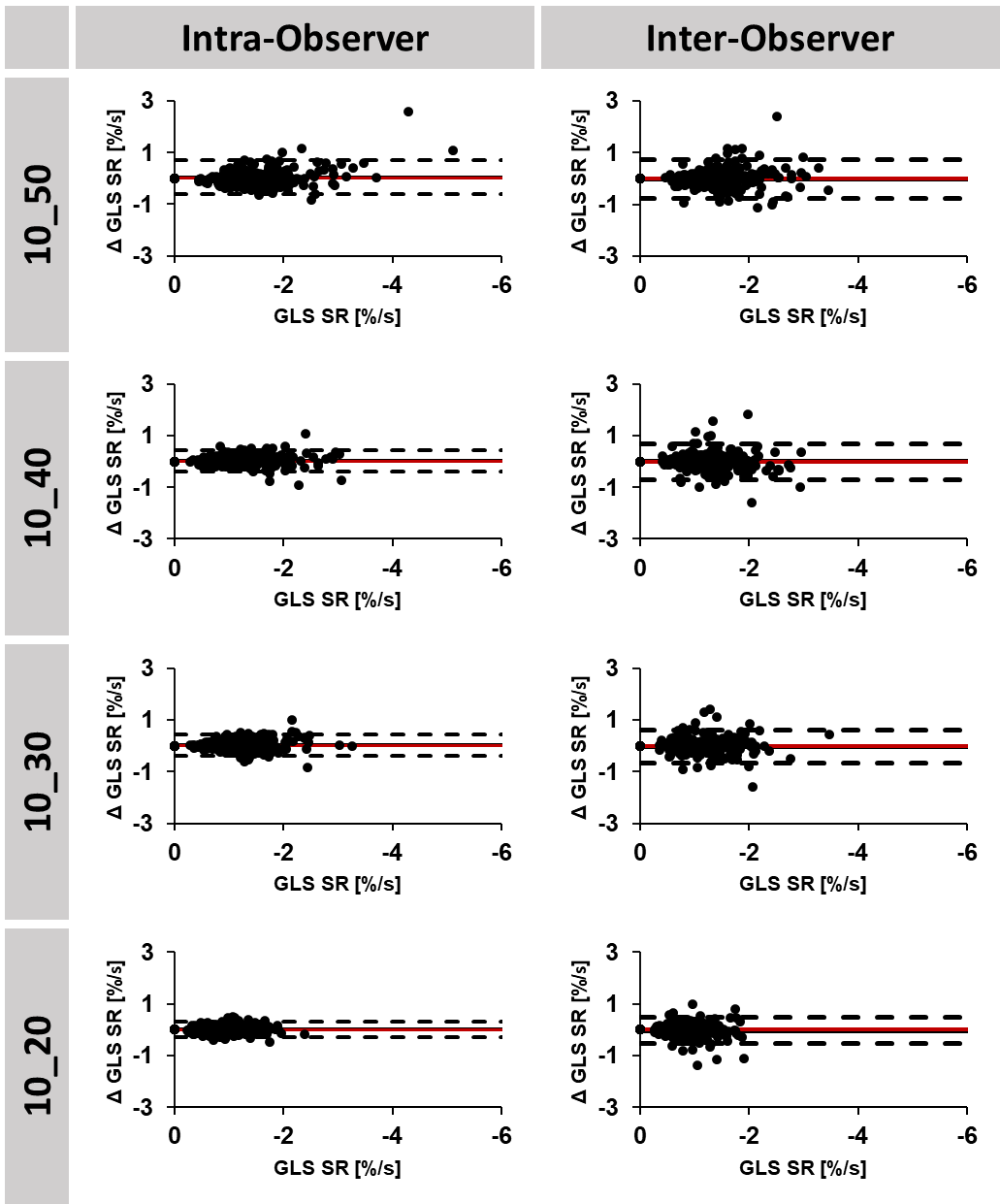


**Figure 7S Agreement of FT-GLS SR depending on Resolution (10 mm and 20-50 frames/cardiac cycle)**

Bland Altman plots are shown for intra- and inter-observer reproducibility of left ventricular global longitudinal strain rate (GLS SR) obtained by Feature-Tracking (FT) depending on spatial (3.0x3.0 mm in plane and 10 mm through-plane) and temporal (20-50 frames/cardiac cycle) resolution. Δ= difference for intra-observer (observer 1 first measurement – second measurement) or inter-observer (observer 1 first measurement – observer 2 measurement) measurements, respectively. Reference for 0 difference in red. (n=252 data points).


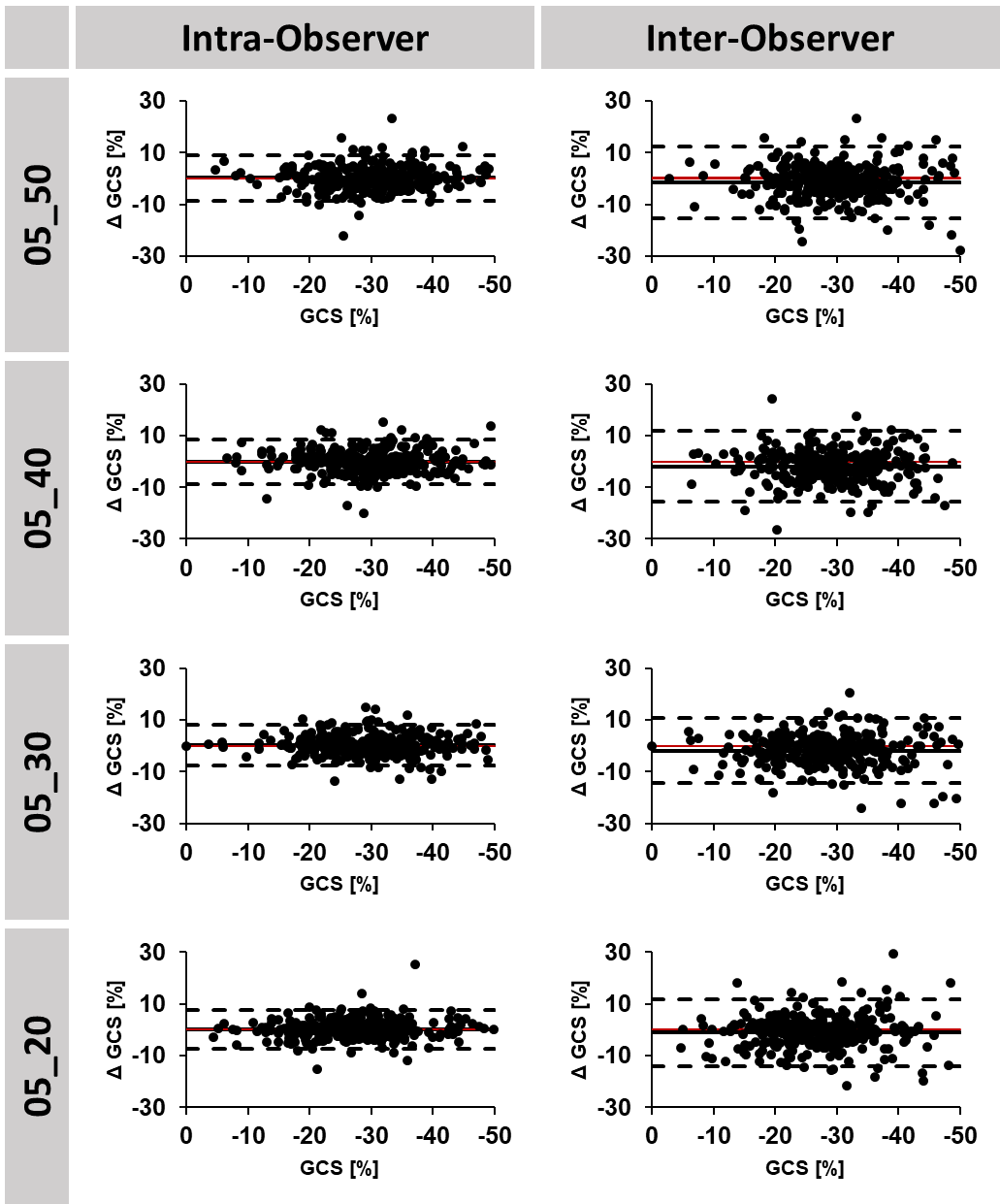


**Figure 8S Agreement of FT-GCS depending on Resolution (5 mm and 20-50 frames/cardiac cycle)**

Bland Altman plots are shown for intra- and inter-observer reproducibility of left ventricular global circumferential strain (GCS) obtained by Feature-Tracking (FT) depending on spatial (1.5x1.5 mm in plane and 5 mm through-plane) and temporal (20-50 frames/cardiac cycle) resolution. Δ= difference for intra-observer (observer 1 first measurement – second measurement) or inter-observer (observer 1 first measurement – observer 2 measurement) measurements, respectively. Reference for 0 difference in red. (n=336 data points).


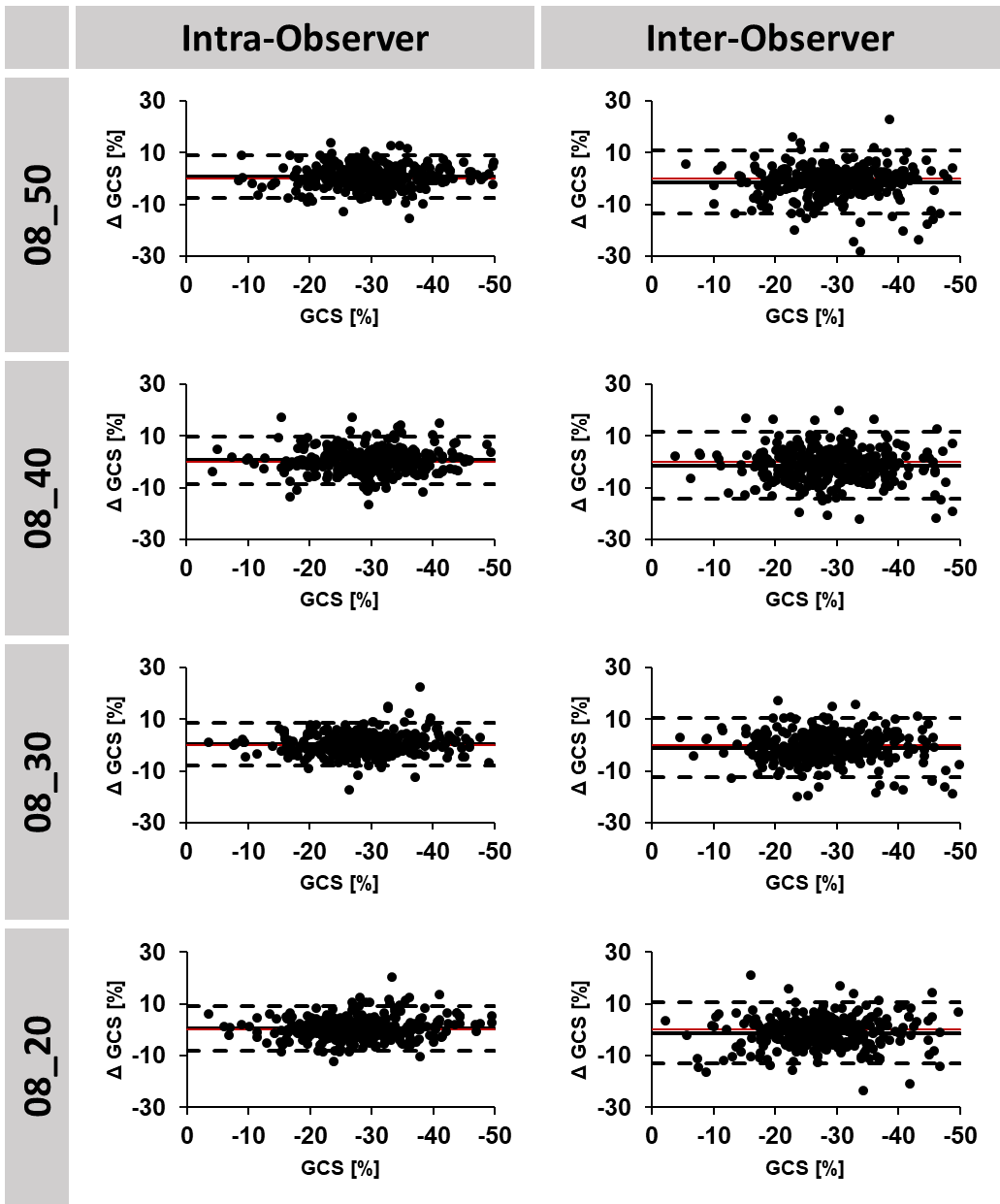


**Figure 9S Agreement of FT-GCS depending on Resolution (8 mm and 20-50 frames/cardiac cycle)**

Bland Altman plots are shown for intra- and inter-observer reproducibility of left ventricular global circumferential strain (GCS) obtained by Feature-Tracking (FT) depending on spatial (1.8x1.8 mm in plane and 8 mm through-plane) and temporal (20-50 frames/cardiac cycle) resolution. Δ= difference for intra-observer (observer 1 first measurement – second measurement) or inter-observer (observer 1 first measurement – observer 2 measurement) measurements, respectively. Reference for 0 difference in red. (n=336 data points).


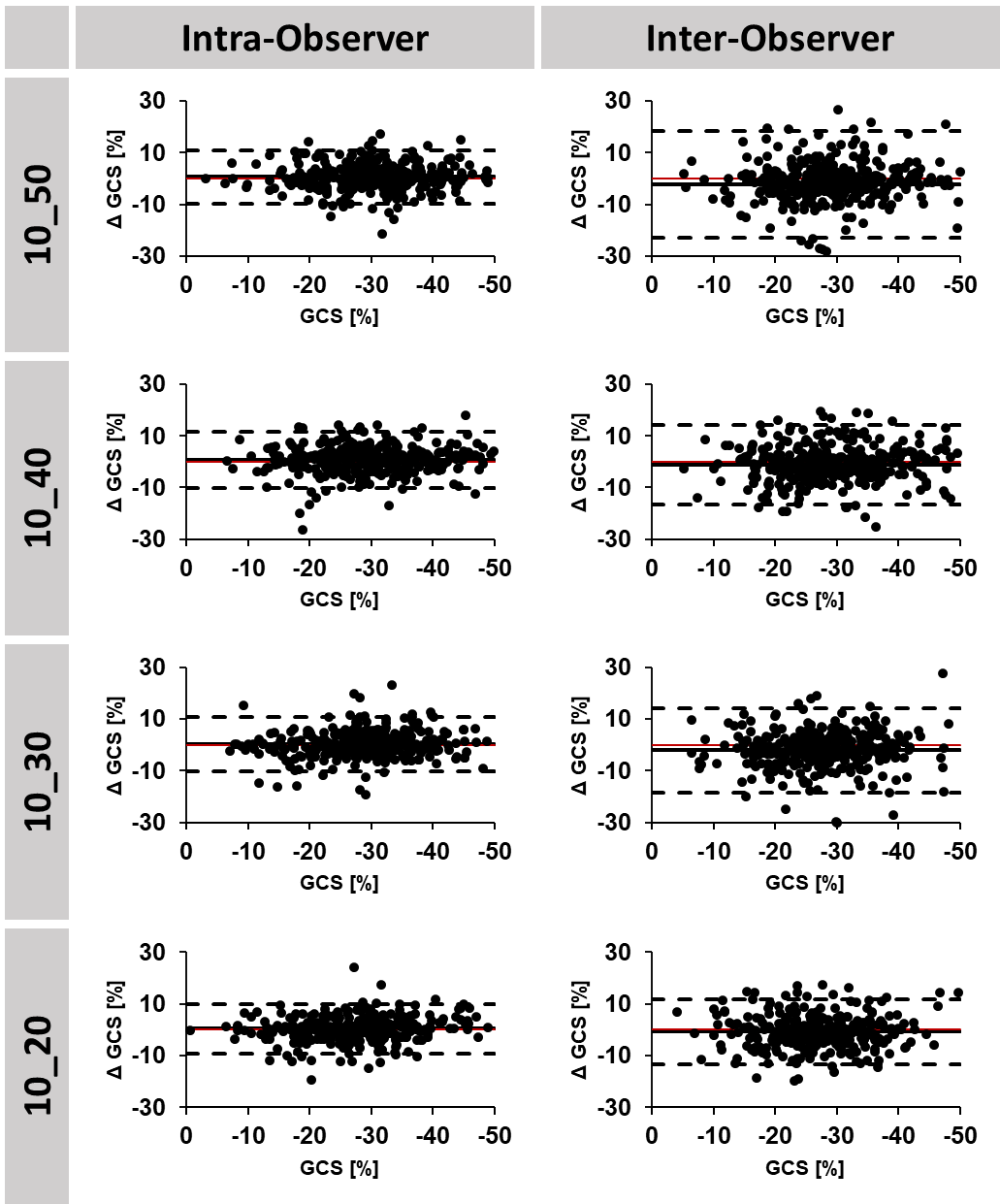


**Figure 10S Agreement of FT-GCS depending on Resolution (10 mm and 20-50 frames/cardiac cycle)**

Bland Altman plots are shown for intra- and inter-observer reproducibility of left ventricular global circumferential strain (GCS) obtained by Feature-Tracking (FT) depending on spatial (3.0x3.0 mm in plane and 10 mm through-plane) and temporal (20-50 frames/cardiac cycle) resolution. Δ= difference for intra-observer (observer 1 first measurement – second measurement) or inter-observer (observer 1 first measurement – observer 2 measurement) measurements, respectively. Reference for 0 difference in red. (n=336 data points).


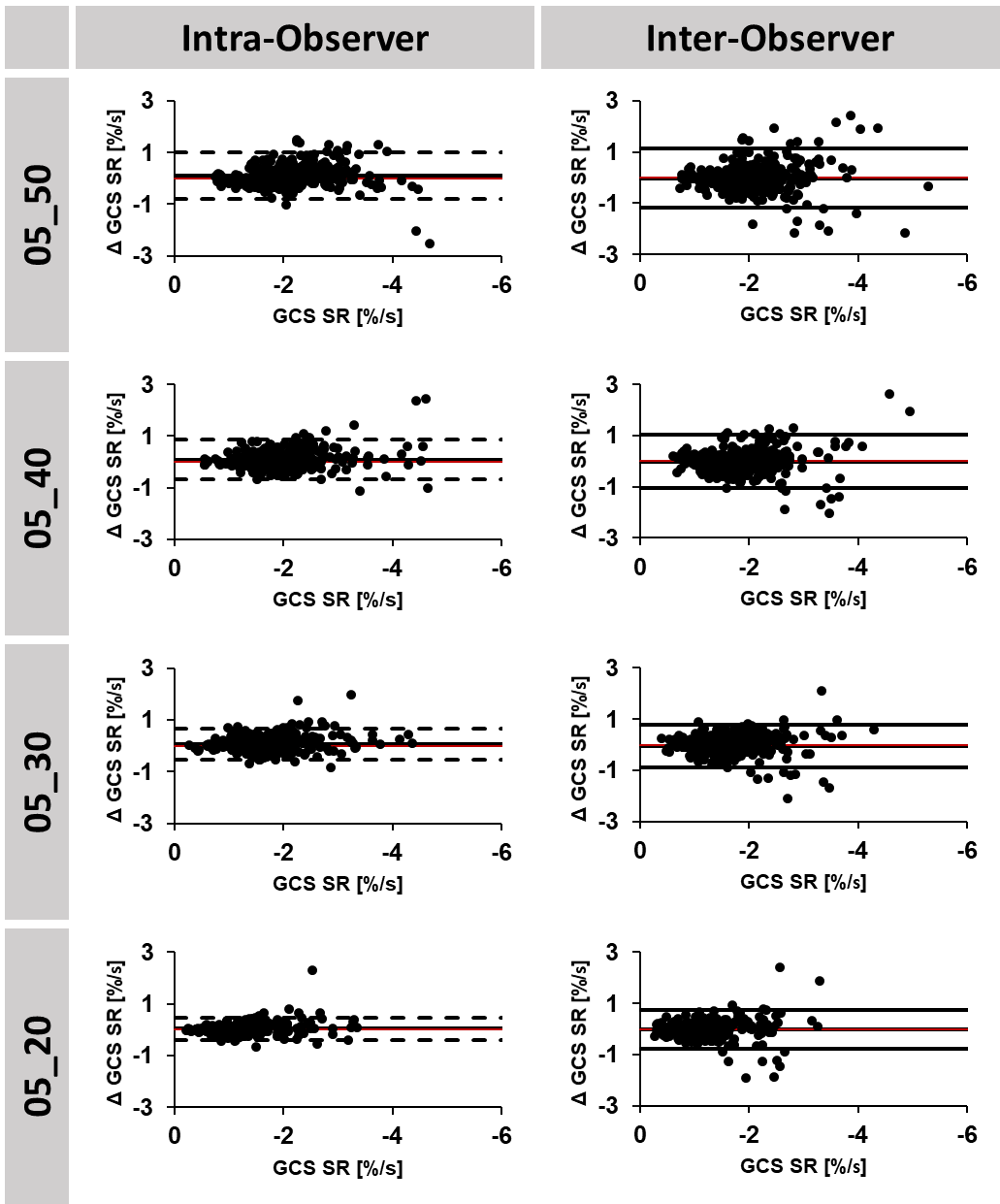


**Figure 11S Agreement of FT-GCS SR depending on Resolution (5 mm and 20-50 frames/cardiac cycle)**

Bland Altman plots are shown for intra- and inter-observer reproducibility of left ventricular global circumferential strain rate (GCS SR) obtained by Feature-Tracking (FT) depending on spatial (1.5x1.5 mm in plane and 5 mm through-plane) and temporal (20-50 frames/cardiac cycle) resolution. Δ= difference for intra-observer (observer 1 first measurement – second measurement) or inter-observer (observer 1 first measurement – observer 2 measurement) measurements, respectively. Reference for 0 difference in red. (n=336 data points).


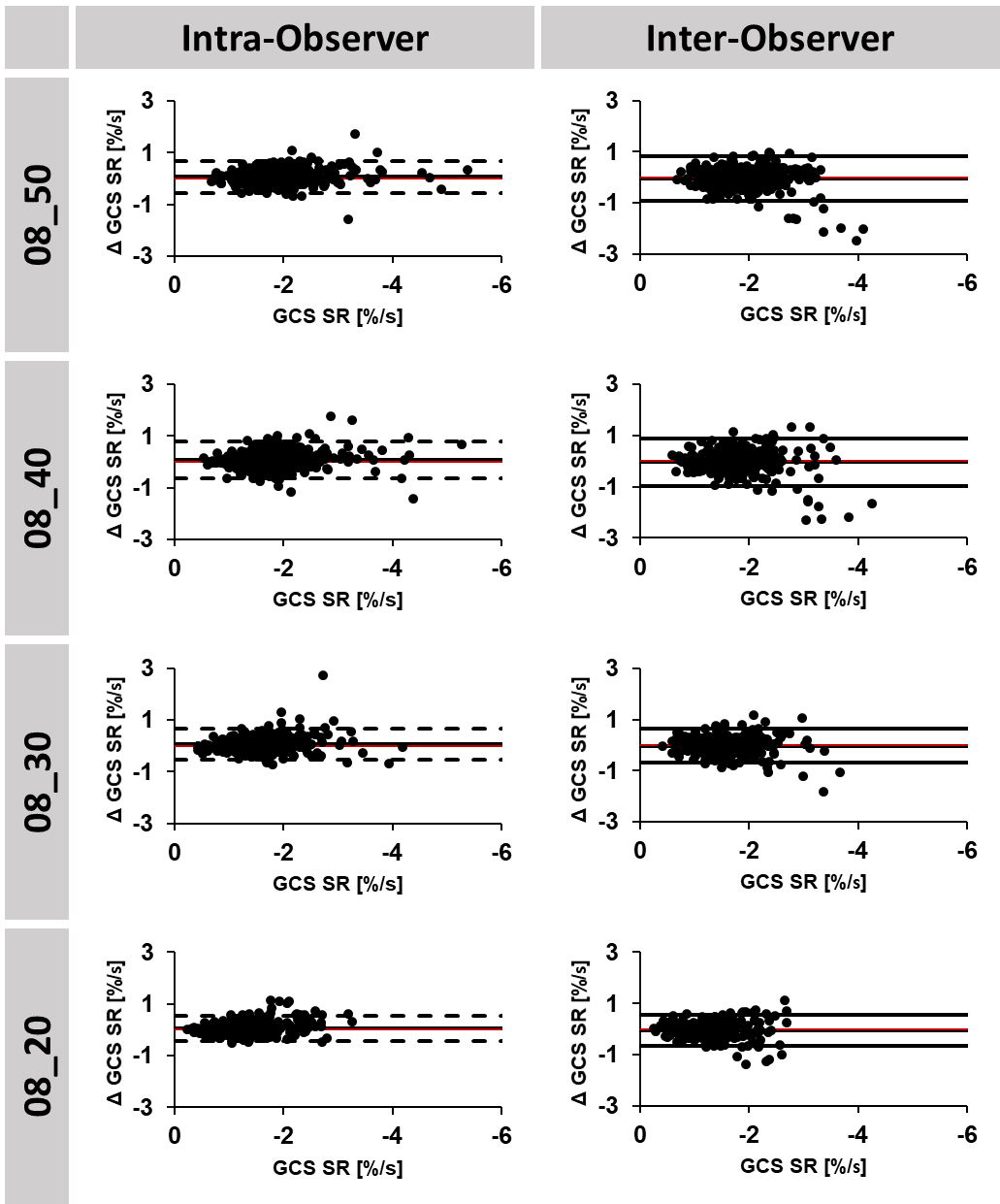


**Figure 12S Agreement of FT-GCS SR depending on Resolution (8 mm and 20-50 frames/cardiac cycle)**

Bland Altman plots are shown for intra- and inter-observer reproducibility of left ventricular global circumferential strain rate (GCS SR) obtained by Feature-Tracking (FT) depending on spatial (1.8x1.8 mm in plane and 8 mm through-plane) and temporal (20-50 frames/cardiac cycle) resolution. Δ= difference for intra-observer (observer 1 first measurement – second measurement) or inter-observer (observer 1 first measurement – observer 2 measurement) measurements, respectively. Reference for 0 difference in red. (n=336 data points).


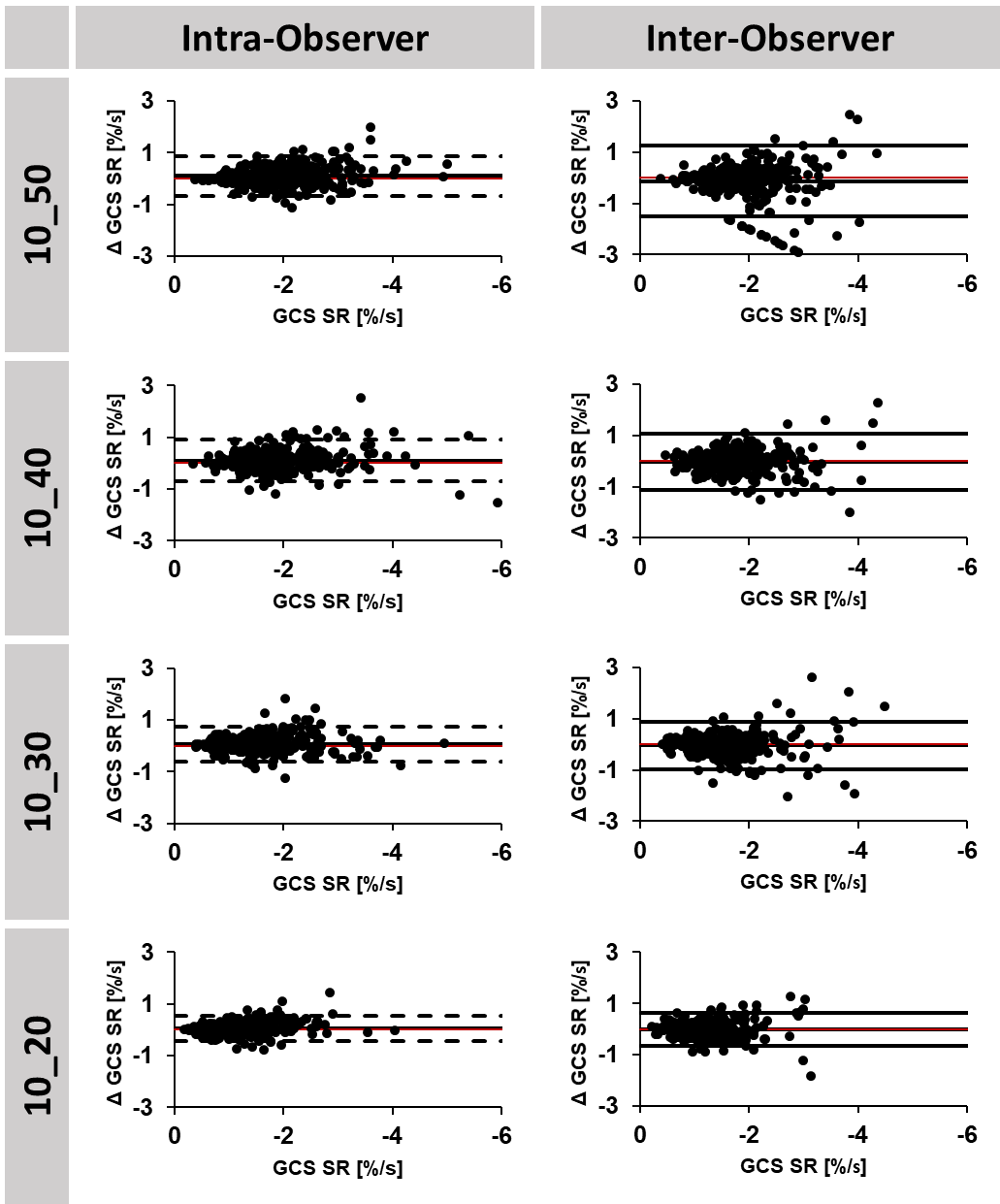


**Figure 13S Agreement of FT-GCS SR depending on Resolution (10 mm and 20-50 frames/cardiac cycle)**

Bland Altman plots are shown for intra- and inter-observer reproducibility of left ventricular global circumferential strain rate (GCS SR) obtained by Feature-Tracking (FT) depending on spatial (3.0x3.0 mm in plane and 10 mm through-plane) and temporal (20-50 frames/cardiac cycle) resolution. Δ= difference for intra-observer (observer 1 first measurement – second measurement) or inter-observer (observer 1 first measurement – observer 2 measurement) measurements, respectively. Reference for 0 difference in red. (n=336 data points).
